# Antithrombotic effect and plasma pharmacochemistry of *Justicia Procumbens* L

**DOI:** 10.1371/journal.pone.0321023

**Published:** 2025-04-30

**Authors:** Hui Zhang, Yating Liu, Fengxian Hu, Siyi Chen, Zhoutao Xie, Hezhen Wu, Ting Zhang, Bo Liu

**Affiliations:** 1 School of Pharmacy, Hubei University of Chinese Medicine, Wuhan, China,; 2 Key Laboratory of Traditional Chinese Medicine Resources and Chemistry of Hubei Province, Wuhan, China,; 3 Modern Engineering Research Center of Traditional Chinese Medicine and Ethnic Medicine of Hubei Province, Wuhan, China,; 4 Hubei Shizhen Laboratory, Wuhan, China,; 5 Hubei Provincial Hospital of Traditional Chinese Medicine, Wuhan, China,; 6 Hubei Provincial Hospital of Integrated Chinese & Western Medicine, Wuhan, China,; 7 Affiliated Hospital of Hubei University of Chinese Medicine, Wuhan, China; Helwan University, EGYPT

## Abstract

**Importance:**

*Justicia procumbens* L. contains lignans with remarkable antiplatelet aggregation effects. Its ethyl acetate extract has demonstrated the efficacy of inhibiting platelet aggregation in preliminary tests. Identifying key components, elucidating the antithrombotic mechanism, and evaluating its potential for treating thrombotic diseases are of great significance.

**Methods:**

Chemical analysis was conducted on the ethyl acetate extract of *J. procumbens*. Animal models of carotid artery and venous thrombosis, combined with proteomics analysis, were employed to study the antithrombotic mechanism.

**Results:**

Chemical analysis identified justicidin B (JB) and chinensinaphthol methyl ether (CME) as the major active antithrombotic constituents. The extract inhibited platelet aggregation by regulating relevant signaling pathways, showing potential in preventing both venous and arterial thrombosis.

**Conclusions:**

This study confirmed that the ethyl acetate extract of *J. procumbens*, especially the lignan components JB and CME, can effectively inhibit platelet aggregation, showing promising prospects in treating thrombotic diseases. This provides ideas for the development of health products based on *J. procumbens* and lays a foundation for further clinical exploration.

## Introduction

Cardiovascular disease is the leading cause of death worldwide and cerebrovascular diseases are an increasingly significant public health issue, imposing a heavy burden on national healthcare systems [1]. Thrombosis is an important factor in cardiovascular disease. Antithrombotic drugs had a huge market of 48.52 billion USD in 2023 and are expected to grow at a compound annual growth rate of 8.1% until 2028. Clinically, antiplatelet drugs and anticoagulants can inhibit thrombosis, reduce the incidence and mortality of acute myocardial infarction, and are effective in preventing thrombotic diseases such as acute coronary syndrome [2]. However, no currently available drugs have both anticoagulant and antiplatelet aggregation effects.

*Justicia procumbens* L., a small flowering plant in the Acanthaceae family, has many varieties and different morphologies with high ornamental value and it is commonly used in urban greening, horticulture, and gardening. The leaves of *J. procumbens* are rich in nutrients, and the plant has a wide range of traditional applications worldwide. The nutrients in its leaves and rhizomes have antioxidant effects and are used to make anti-aging and antioxidant products with diverse applications in the functional food, cosmetic, and pharmaceutical industries. In India, the leaves are made into a soup to treat asthma [3], and in Nepal, a soup of the roots is used to treat typhoid fever [4]. In China, the whole *J. procumbens* herb is used to alleviate pharyngolaryngitis, relieve pain, and promote blood circulation [5]. *Jianer* syrup and *Fufukang* spray, two compound preparations containing *J. procumbens,* are marketed in China [6], and an anti-asthma extract preparation of *J. procumbens* is completing phase I clinical trials in Korea [7]. The constituents of *J. procumbens* have antioxidant, anti-inflammatory, antibacterial, antitumor, antiviral [8], anti-human immunodeficiency virus-1 [9], and analgesic [10] effects, and inhibit nephritic cell proliferation. Malaysians eat *J. procumbens* as food during famines. In China, some people make *J. procumbens* and Jujube into Jue Chuang Hong Zao soup.

In this study, we screened the effective extract of *J. procumbens* (Effective extract: a specific group of compounds within *J. procumbens*, possessing antiplatelet aggregation activity and being distinct from ethyl acetate extract) to detect its antiplatelet aggregation activity and determined the content of the main compounds. The findings of this study provide a scientific basis for the development of drug and functional foods using *J. procumbens* as a raw material to prevent thrombosis.

## Materials and methods

### Instruments

The instruments used in this study include the Vanquish F high-performance liquid chromatograph, Q-Exactive HF-X MS/MS (ThermoFisher Scientific, USA), Agilent 1200 Liquid Chromatograph system (Agilent Company, USA), AL204 analytical balance, XPE26 microanalytical balance (Mettler Toledo Instruments Shanghai Co., Ltd., China), LNG-T98 refrigerant centrifugal concentration dryer, LNG-T88 benchtop rapid centrifugal concentration dryer (Taicang Huamei Biochemical Instrument Factory, Ltd., China), Rotavapor R-300 rotary evaporators (Buchi Laboratory Equipment Co., Ltd., Switzerland), DLSB0–5/20 cryogenic cooling circulating pump (Zhengzhou Great Wall Technology Industry and Trade Co., Ltd., China), ZF-6 triple-use ultraviolet analyzer (Shanghai Jiapeng Technology Co., Ltd., China), DW-86W150 medical low temperature freezer (-80 °C) (Aucma Co., Ltd., China), LBY-NJ4 Whole Blood Aggregometer (Techlink Biomedical, China), ACQUITY ultra-performance liquid chromatography (UPLC) H-Class system (Waters, USA), Waters Xevo G2-XS QTOF system, Waters Alliance e2695 (USA), JB-P5 Embedding Machine (Wuhan Junjie Electronics Co., Ltd., China), RM2016 Pathological Microtome (Shanghai Leica Instruments Co., Ltd., China), JB-L5 Freezing Stage (Wuhan Junjie Electronics Co., Ltd.), KD-P Tissue Spreading Machine (Kedi Instrument and Equipment Co., Ltd., Jinhua City, Zhejiang Province, China), DGX-9003B oven (Shanghai Fuma Experimental Instrument Co., Ltd., China), 10212432C slides and coverslips (Jiangsu Shitai Experimental Equipment Co., Ltd., China), Nikon Eclipse Ti-SR Inverted Fluorescence Microscope (Nikon, Japan), and the Nikon DS-U3 Imaging System (Nikon), Eclipse XDB-C18 Column (Agilent Technologies), ProteomeDiscoverTM (Thermo Fisher Scientific).

### Plants, animals, and reagents

#### The source of *J. procumbens.*

*J. procumbens* herb was collected from Wuchang District, Wuhan City, Hubei Province, China, and identified by Professor Liu Hegang of Hubei University of Chinese Medicine. A voucher specimen (JC20221002) was registered at the College of Pharmacy, Hubei University of Chinese Medicine.

#### Animals.

Sixty-four specific pathogen-free Sprague-Dawley (SD) rats, 6 weeks old, 44 males and 20 females, weighing 220 ± 20 g, were purchased from the Experimental Animal Center of China Three Gorges University [license No. SCXK(E)2017–0012]. The animals were kept at a temperature of 20–25°C, 50 ± 10% humidity, with free access to diet and water.

All experimental procedures were approved by the Animal Research Committee of Hubei University of Chinese Medicine (Hubei, China; approval No. HUCMS202006012).

#### Reagents.

The other materials used in this study included *J. procumbens* ethyl acetate extract*,* effective extract, justicidin B (JB), chinensinaphthol methyl ether (CME), and neojusticin B (NJB) prepared in our laboratory, aspirin enteric-coated tablets (Shi Yao Group Ouyi Pharmaceutical Co., Ltd., China, BN:286200283), pentobarbital sodium salt (Merck Drugs & Biotechnology, Germany, BN:P11011), 3.2% sodium citrate anticoagulant blood collection tubes (Jiangsu Kangjie Medical Devices Co., Ltd., China), 2 mL trisodium citrate dihydrate blood collection tubes (Shan Dong Ao Sai Te Medical Devices Co. Ltd., China), acetonitrile, methanol, formic acid (liquid chromatography/mass spectroscopy LC/MS standard, Thermo Fisher Scientific), sodium carboxymethyl cellulose, iodine, FeCl_3_, sodium dodecyl sulfate, paraformaldehyde, ethanol, dimethylbenzene, hydrochloric acid, ammonia, neutral gum, petroleum ether (60–90ºC), ethyl acetate, *N*-butanol, acetone, acetonitrile, dichloromethane, toluene, acetic acid (analytical purity, National Pharmaceutical Group Chemical Reagent Co., Ltd., China), iodoacetamide, triethylammonium bicarbonate buffer TEAB (Sigma, Germany), Coomassie brilliant blue staining solution (Beyotime, China), modified trypsin protease, Tandem Mass Tag (TMT) 10Plex, protein ladder, protease inhibitor cocktail, Pierce BCA protein assay kit, NuPAGE 10% BT gel 1.0 mm 12 well, Bond-Breaker TCEP Solution (ThermoFisher Scientific), 100–200 column chromatography silica gel, thin-layer chromatographic silica gel G (Qingdao Haiyang Chemical Co., Ltd., China), rivaroxaban (Meilunbio, China‌), ceftriaxone sodium (Shanghai yuanye Bio-Technology Co., Ltd, China), anhydrous ethanol (Sinopharm Chemical Reagent Co., Ltd., China), xylene (Sinopharm Chemical Reagent Co., Ltd.), hematoxylin staining solution (Servicebio, China), hydrochloric acid (Sinopharm Chemical Reagent Co., Ltd.), ammonia water (Sinopharm Chemical Reagent Co., Ltd.), eosin staining solution (Servicebio), and neutral gum (Sinopharm Chemical Reagent Co., Ltd.).

### Multi-database analysis of *J. procumbens* for treating thrombotic diseases

#### Prediction of compound targets and disease targets.

An in-depth screening and analysis of the effective components of *J. procumbens* were conducted based on a high-throughput experiment- and reference-guided database of traditional Chinese medicine (HERB, http://herb.ac.cn/) and the PubChem database (https://pubchem.ncbi.nlm.nih.gov/), which yielded a total of 39 compounds. The SMILES format of these compounds was obtained from the PubChem database. The potential targets for these compounds were then obtained from HitPick (http://mips.helmholtz-muenchen.de/hitpick/), similarity ensemble approach (SEA, http://sea.bkslab.org/), and Target Hunter of Small Molecule (TargetHunter) databases (http://www.cbligand.org/targethunter) [11]. These targets were integrated, and duplicate targets were removed for network pharmacology research.

Disease targets were obtained from the Genecards database (https://www.genecards.org/). A search using the keyword “platelet aggregation” yielded 8536 potential targets related to platelet aggregation-associated diseases.

#### Construction of a protein-protein interaction network.

The target information of the 39 compounds was intersected with the potential targets related to platelet aggregation-associated diseases, and common component-disease targets were obtained. These common targets were imported into the STRING database (https://string-db.org/) [12] to construct a protein-protein interaction (PPI) network. This network was then reconstructed and visualized using Cytoscape 3.10.1 (https://cytoscape.org/) [13]. A network topology analysis was conducted to filter out the top 20 key targets by activating the CytoNCA plugin in the software and using degree centrality (DC) as the measurement standard.

#### Enrichment analysis of targets.

The component-disease common targets were imported into the DAVID database (https://david.ncifcrf.gov/) [14] for gene ontology (GO) functional enrichment analysis and Kyoto Encyclopedia of Genes and Genomes (KEGG) pathway enrichment analysis. The GO functional enrichment analysis mainly includes three components: biological processes (BPs), cellular components (CCs), and molecular functions (MFs). The antiplatelet aggregation function and key mechanisms of the effective components can be assessed by analyzing these three components. Key signaling pathways were screened using KEGG pathway enrichment analysis based on the proportion of genes in each pathway and P-value information.

#### Construction of a disease-pathway-target-component network.

A disease-pathway-target-component (D-P-T-C) network was constructed based on the known 39 compounds and the results of KEGG pathway enrichment analysis in Cytoscape3.10.1 to further ascertain the correlation between the potential effective components of *J. procumbens* and diseases related to platelet aggregation (PA), as well as the associations among components, targets, and pathways. The interactive network was analyzed and filtered based on degree values. Compounds with high degree values often have more effective potential targets for diseases related to PA, and targets affecting multiple key signal pathways can be obtained at the same time.

### Analysis of blood components after gavage with ethyl acetate from *J. procumbens*

#### Chemical composition analysis of the ethyl acetate extract of *J. procumbens.*

#### Preparation of the ethyl acetate extract.

Dry, powdered *J. procumbens* herb (15 kg) was sieved through a sieve No. 2 sieve, wetted with 80% ethanol, and loaded into a percolation bucket. After percolation with 80% ethanol (150 L), the percolated solution was concentrated under reduced pressure to remove the alcohol and was extracted with ethyl acetate (4 L). Concentration and drying under reduced pressure yielded 90 g of ethyl acetate extract.

#### Preparation of the effective extract.

The ethyl acetate extract was dissolved in methanol to form a runny paste, and an appropriate amount of silica gel (100–200 mesh) was added with stirring. The slurry was dried at 40ºC under reduced pressure, and the pre-adsorbed extract was ground through a No. 6 sieve. For column chromatography, silica gel (2 Kg, 100–200 mesh) was prepared with petroleum ether-acetone (2:1). The pre-adsorbed extract was added to the top of the column and eluted with petroleum ether-ethyl acetate (4:1, 8 L). The eluate was collected and concentrated to dryness to provide 15 g of *J. procumbens* effective extract.

#### Separation of compounds.

Compounds were separated from the effective extract using high-performance liquid phase preparative chromatography. An acetonitrile-water system was used for isocratic elution to prepare JB (87 mg), CME (52 mg), and NJB (27 mg). The structures of these compounds were elucidated from their MS and nuclear magnetic resonance spectral data.

#### Analysis of constituents entering the bloodstream from the ethyl acetate extract.

#### Sample preparation.

Ethyl acetate extract: Ethyl acetate extract powder (5 mg) was accurately weighed and placed in a 10 mL volumetric flask. Then, 10 mL methanol was added, and the mixture was extracted using an ultrasonic bath (50 Hz) for 10 min. The extract was filtered through a 0.22 μm membrane filter.

Plasma sample preparation: SD rats in the blank group were orally administered carboxymethyl cellulose sodium (CMC-Na) (0.75 mL/100 g), and ethyl acetate extract rats were orally administered ethyl acetate extract (7.5 mg/100 g). After 1.5 hours, abdominal aortic blood was collected, plasma was separated, and five times the volume of acetone was added. The mixture was vortexed and shaken at 4000 rpm for 10 minutes and then centrifuged. The upper clear liquid was centrifuged at 12,000 rpm/min for 10 minutes and the supernatant was removed for UPLC-MS analysis. The plasma was filtered through a 0.22 μm membrane filter.

#### UPLC-MS conditions.

Liquid phase conditions: Chromatographic separation was conducted using a Waters ACQUITY BEH C18 column (1.7 μm, 100 × 2.1 mm). Water served as mobile phase A, while acetonitrile was used as mobile phase B. The gradient elution program was: 0–20 min, 15%–35% B, and 20–30 min, 35%–50% B. The flow rate was 0.3 mL/min, and the injection volume was 2 μL. The column temperature was maintained at 30°C.

Mass spectrometry conditions: An electrospray ionization (ESI) source was employed to collect data in both the positive and negative ion modes. The mass spectrometry-enhanced resolution mode was utilized for acquiring MS^1 and MS^2 spectra over a scan range of m/z 50–1500 Da. The electrospray voltage was set to 3000 V, the cone voltage to 20 V, and the ESI source temperature was kept at 100°C. The curtain gas flow rate was 50 L/h, and the desolvation gas (N_2_) flow rate was 1000 L/h.

### Determination of the content of the effective extract of *J. procumbens*

#### Preparation of sample solution and mixed control solution.

The effective extract was prepared as a 0.480 mg/mL solution using the method used to prepare the ethyl acetate extract sample solution. A mixed control solution of JB, CME, and NJB was prepared in methanol at concentrations of 0.162 mg/mL, 0.141 mg/mL, and 0.132 mg/mL, respectively.

#### Chromatographic conditions.

High-performance liquid chromatography (HPLC) was performed using an Eclipse XDB-C18 column (4.6 mm × 150 mm, 5 μm) and gradient elution of mobile phase A (water) and B (acetonitrile) of 0 min, 55% A; 8 min, 55% A; 12 min, 35% A; and 20 min, 35% at a flow rate of 0.6 mL/min. The injection volume was 10 μL, the column temperature was 30ºC, the detection wavelength was 256 nm, and the run time was 20 min.

### Antiplatelet aggregation tests of ethyl acetate extract, effective extract, JB, and CME

Blood was drawn from SD rats into plastic tubes and anti-coagulated with 3.2% sodium citrate (the anticoagulant to blood ratio was 1:9), then centrifuged at 800 rpm for 8 min, and the upper layer of platelet-rich plasma (PRP) was separated. The lower layer was centrifuged again at 3500 rpm, and the upper layer was taken as platelet-poor plasma (PPP).

PRP and PPP were collected from the model, aspirin, ethyl acetate extract, effective extract, JB, and CME groups. The PRP platelet count was adjusted to 3 × 10^8^ platelets/mL with PPP. In all experiments, 260 μL of PRP (3 × 10^8^ platelets/mL) and 30 μL of the test compound solution were incubated at 37ºC for 25 min while stirring, magnetic stirring was turned on immediately after adding the inducer, and 10 μL of each of the different inducing agents was added (0.13 mg/mL adenosine diphosphate (ADP), 7.18 mg/mL arachidonic acid (AA), 0.28 mg/mL 5-hydroxytryptamine (5-HT), 0.19 mg/mL platelet-activating factor (PAF), and 10 IU/mL thrombin). The maximum platelet aggregation rate was determined within 5 min by adding 10 μL of different inducers (0.13 mg/mL ADP, 7.18 mg/mL AA, 10 IU/mL thrombin, 0.28 mg/mL 5-HT, and 0.19 mg/mL PAF) and adjusting to zero with a parallel set of PPP reaction tubes and adjusting to 100% with PRP reaction tubes [15].

### Clot retraction

Fresh blood from SD rats was collected and placed into sodium citrate blood collection tubes. PRP was then separated, and the platelet concentration was adjusted to approximately 3 × 10^8^ cells/mL. For the coagulation initiation, 100 μL of PRP was added to each well of a 96 - well plate, followed by the addition of 10 μL of thrombin solution (1 U/mL) and 10 μL of calcium chloride solution (0.2 M) to start the coagulation process. A control group was set up simultaneously, which only contained PRP, thrombin, and calcium chloride. Regarding the drug treatment, immediately after the coagulation was initiated, different concentrations of the test drugs, namely the ethyl acetate extract (0.10 mg/mL), JB (150 μM), CME (30 μM), along with a reference drug Aspirin (70 μM) and the effective extract (0.15 mg/mL), were added to each well and gently mixed. For the observation of clot retraction, the 96 - well plate was incubated at 37 °C, and the clot retraction was observed at different time points of 1 hour, 2 hours, 3 hours, and 4 hours. The change in absorbance at a wavelength of 650 nm was measured using a microplate reader to indirectly reflect the clot retraction.

### Antithrombotic effects of the effective extract on FeCl_3_-induced carotid artery thrombosis in SD rats

#### Animal groups.

Forty SD rats, 20 males and 20 females, fed a normal diet were observed to have no abnormal reactions after a one-week acclimatization period. The rats were kept in plastic cages with free access to food and water. They were maintained in a controlled environmental temperature of 20°C, 50–60% relative humidity, and 12 h of light per day. They were divided into five groups (model group, aspirin group, high, medium, and low-dose extract groups) with eight animals randomly assigned to each group.

#### Methods of administration.

After fasting for 8 h, the model group was administered 0.5% CMC-Na solution (0.75 mL/100 g) by gavage, the aspirin group and high, medium, and low-dose effective extract groups were gavaged (0.75 mL/100 g) with the corresponding concentration of drug (high-dose extract: 2.02 mg/mL, medium-dose extract: 1.51 mg/mL, and low-dose extract: 1.00 mg/mL).

#### Establishment of an SD rat carotid artery thrombosis model using FeCl_3_ induction.

After gavaging, each animal was administered 2% pentobarbital sodium solution (0.25 mL/100 g) by intraperitoneal injection over 5 min. After anesthesia, the rats were fixed in the supine position, and the surgical area was dehaired and disinfected. The skin was cut longitudinally above the cricothyroid cartilage along the middle of the neck, and the connective tissues were bluntly separated with hemostatic forceps to expose the carotid muscles and the carotid arteries along the muscle gap. The carotid artery was exposed and freed for about 3 cm. A 1.0 cm-wide sealing tape was used to isolate the carotid artery, and then a strip of filter paper (0.6 cm wide) soaked in 20% FeCl_3_ solution was wrapped around the carotid artery segment to separate the sample. The strip of filter paper was removed after 15 min, and blood was collected from the abdominal aorta after 40 min into a blood collection vessel containing 3.2% sodium citrate. The wet thrombus-containing vessel was accurately weighed. The corresponding carotid artery on the other side was taken as a control, and the blood remaining in the vessel was drained with clean and dry filter paper [16].

#### Hematoxylin and eosin staining method.

The carotid artery on the modeling side and the control side vessels of the SD rats in each group were embedded in paraffin, sectioned, and stained with hematoxylin and eosin (H&E). First, the fresh vascular tissues from these sides were fixed in 4% paraformaldehyde for over 24 hours. After removal, the relevant parts were trimmed with a scalpel and placed in dehydration boxes with the corresponding labels. Then, the dehydration boxes with the tissues were put in the dehydrator’s hanging baskets for sequential dehydration in different concentrations of alcohol. Next, the wax-soaked tissues were placed in an embedding machine. Melted wax was poured into the embedding frame, and when the wax was about to solidify, the tissues were removed, placed as required and labeled. After cooling at -20°C, the wax blocks were removed and trimmed. Finally, the trimmed wax blocks were cut into 4 μm-thick slices on a paraffin microtome. The slices were flattened in 40°C water and then baked in a 60°C oven.

The sliced sections were stained with H&E. The slices were first treated with xylene, anhydrous ethanol, and different concentrations of alcohol and then washed with water to achieve dewaxing to water. Subsequently, the slices were immersed in Harris hematoxylin for 3–8 minutes, followed by washing with tap water, differentiating with 1% hydrochloric acid-alcohol for a few seconds, washing again, bluing with 0.6% ammonia water, and finally rinsing with running water to stain the cell nuclei. Then, the slices were immersed in an eosin staining solution for 1–3 minutes to stain the cytoplasm. Finally, the slices were dehydrated and made transparent with various alcohols and xylene, dried in the air, and mounted with neutral gum. These procedures allowed the tissue morphology and other aspects of the carotid artery model side and control side vessels to be observed and analyzed.

### Proteomic analysis of platelets for the effective extract

Part of the PRP was centrifuged at 3500 rpm for 8 min. The precipitate was washed 2–3 times with 100 µ L of isotonic phosphate buffer at pH 7.4, centrifuged at 3500 rpm for 8 min, and the supernatant was removed. The precipitate platelet samples were transferred to Eppendorf tubes and stored at -80°C.

The appropriate amount of frozen platelet sample was transferred to an Eppendorf tube, and the appropriate amount of protein lysate (8 M urea, containing protease inhibitors) was added, sonicated on ice for 2 min, and lysed on ice for 30 min, during which time it was vortexed and mixed for 5–10 s at 5 min intervals. After centrifugation at 4°C for 30 min at 12,000 g, the supernatant was collected, and the protein content was determined using the bicinchoninic acid (BCA) assay.

Lysate was added to a 100 μg protein sample, and final concentrations of 100 mM triethylammonium bicarbonate buffer (TEAB) and 10 mM TCEP were added sequentially, and the mixture was reacted for 60 min at 37°C. Iodoacetamide was added to a final concentration of 40 mM, and the reaction continued for 40 min at room temperature in the dark. Pre-cooled acetone (acetone:sample 6:1 v/v) was added to each tube, and after 4 h at -20°C, it was centrifuged at 10,000 g for 20 min, and the precipitate was separated. The sample was fully dissolved in 100 µ L of 100 mM TEAB, trypsin was added at a mass ratio of 1:50 (enzyme:protein), and digestion was carried out overnight at 37°C. Peptides were quantified using a ThermoFisher Scientific Peptide Quantification Kit. TMT quantification was followed by reverse-phase liquid chromatography one-dimensional separation using 2% acetonitrile in ammonia adjusted to pH 10 (phase A) and 80% acetonitrile in ammonia adjusted to pH 10 (phase B), with gradient elution of 0–1.9 min, 0% B; 1.9–2 min, 0–5% B; 2–17 min, 5–5% B; 17–18 min, 5–10% B; 18–35.5 min, 10–30% B; 35.5–38 min, 30–36% B; 38–39 min, 36–42% B; 39–40 min, 42–100% B; 40–44 min, 100% B; 44–45 min, 100–0% B; and 45–48 min, 0% B. The UV detection wavelength was 214 nm, and the flow rate was 200 mL/min.

Two-dimensional analysis was performed using Nano LC-MS/MS (Easy-nLC 1200 combined with Q Exactive HF-X mass spectrometer). The samples were separated on a C18 column (75 μm x 25 cm, ThermoFisher) at a flow rate of 300 nL/min using 2% acetonitrile plus 0.1% formic acid (phase A) and 80% acetonitrile plus 0.1% formic acid (phase B) with gradient elution of 0–50 min, 8–23% B; 50–57 min, 23–29% B; 57–60 min, 29–38% B; 60–61 min, 38–48% B; 61–62 min, 48–100% B; and 62–90 min, 100% B.

MS and MS/MS acquisitions were automatically switched between MS and MS/MS with mass spectral resolutions of 120,000 and 45,000, respectively. MS was performed with a full sweep (*m/z* 350–1500), and the top 20 parent ions were selected for secondary fragmentation with a dynamic exclusion time of 20 s.

Raw files were analyzed using ProteomeDiscovererTM Software 2.4, and the database used for the search was Uniprot (https://www.uniprot.org). Finally, the data were analyzed on the Majorbio platform (https://www.majorbio.com). The details of proteomics search parameters are presented in S1 File of the supporting information.

### Deep vein thrombosis animal experiment

#### Animal groups.

Twenty-four male SD rats observed to have no abnormal reactions after a one-week acclimatization period were randomly divided into three groups (model group, rivaroxaban group, and effective extract group), with eight animals in each group. They were housed in plastic cages with free access to water and food, maintained at a controlled temperature of 20°C, relative humidity of 50–60%, and 12 hours of light per day.

#### Methods of administration.

After fasting for 8 hours, the model group was administered 0.5% CMC-Na solution (0.75 mL/100 g) by gavage, the rivaroxaban group received 0.1050 mg/100 g by gavage, and the effective extract group was administered 2 mg/mL by gavage. For all groups, the volume of 0.5% CMC-Na solution was 0.75 mL/100 g.

#### Inferior vena cava stenosis model establishment.

Rats were gavaged with the treatments and anesthetized 5 min later with 2% pentobarbital sodium (0.25 mL/100 g). Once anesthetized, the rats were placed in the supine position on a heated pad covered with sterile drapes and secured on the surgical table. Their rectal temperature was maintained at 37 ± 0.5°C. The surgical site was shaved and disinfected with povidone-iodine, and a midline abdominal incision was made. After moving some intestines to the right, the inferior vena cava was exposed. The exposed area was covered with warm saline-soaked gauze to prevent dehydration. The inferior vena cava was bluntly dissected and ligated below the left renal vein using a 5–0 non-absorbable silk suture along with an acupuncture needle (0.35 mm × 50 mm). The needle was removed, and all horizontal branches between the left renal vein and the iliolumbar vein were ligated. After ligation, the organs were moistened with saline and returned to the abdominal cavity, and the incision was closed in layers using 5–0 non-absorbable silk sutures. Ceftriaxone sodium powder was applied to each layer to prevent infection. The surgical site was disinfected with povidone-iodine, and after the rats awoke, they were allowed free access to water and normally housed. The abdomen was reopened 24 hours later to remove the thrombus. Twenty-four hours after model establishment, the rats’ abdomens were opened to observe thrombus formation in the inferior vena cava and its branches. Blood samples were taken from both the inferior vena cava (IVC) and the abdominal aorta, and the vessels were dissected after blood collection. The segment of the vessel containing the thrombus, from the site of IVC stenosis to the site of branch ligation, was removed. Surface blood was blotted with filter paper as much as possible, and the wet weight of the thrombus was measured using an analytical balance and recorded [17].

### Molecular docking

The three-dimensional (3D) structures of JB and CME were drawn using ChemBio3D ΜLtra (https://www.perkinelmer.com/category/chembiodraw), energy-optimized using MM2 force field and saved in PDB format. The proteins significantly regulated in the proteomics test were searched in the RCSB PDB database (https://www.rcsb.org/), and the corresponding protein 3D data were downloaded to remove solvents, ions (Na^+^, Ca^2+^, Mg^2+^), and other potential interfering molecules using PyMOL software (https://pymol.org/2/). These proteins have not been reported to have a clear binding active site, which is related to their molecular interactions and stability in performing their corresponding functions. Virtual docking was performed using AutoDock Vina software (http://vina.scripps.edu/) [18], and the hydrogenation of receptor proteins, hydrogenation of ligands and the grid box center coordinates were performed and set by AutoDock Tools. Considering the similar size and shape of the binding pockets in the studied proteins and the comparable dimensions of the ligands, a uniform grid box with the size of 30 × 30 y × 30 z points and a spacing of 1 Å was applied for all the docking simulations. The grid box was designed to fully cover the binding pocket of the receptor protein, with extensional space in each direction to allow for the flexibility and conformational sampling of the ligands. All other parameters are default values. To validate the reliability of the docking results, we searched the literature and the RCSB PDB database for known reported inhibitors of F12 and F9 and their crystal structures. Unfortunately, no F12-related confirmed inhibitors and their crystal structures were found, so we re-docked F9 with its inhibitor (CFM-184, PubChem CID:126507438) reported in the RCSB PDB database and Pubmed (https://pubmed.ncbi.nlm.nih.gov/) with the same parameters and methods as a supporting evidence [19]. Each docking binding energy above was calculated three times to ensure the stability of the results.

### Statistical methods

The data are presented as the mean ± standard error of the mean (SEM). The statistical significance of differences between groups was determined using the Student’s t-test or ANOVA multiple comparisons using GraphPad Prism 9.5 (GraphPad, USA). Differences are significant at P < 0.05.

## Results

### Multi-database analysis of the effective components of *J. procumbens* for treating thrombotic diseases

**Fig 1:**
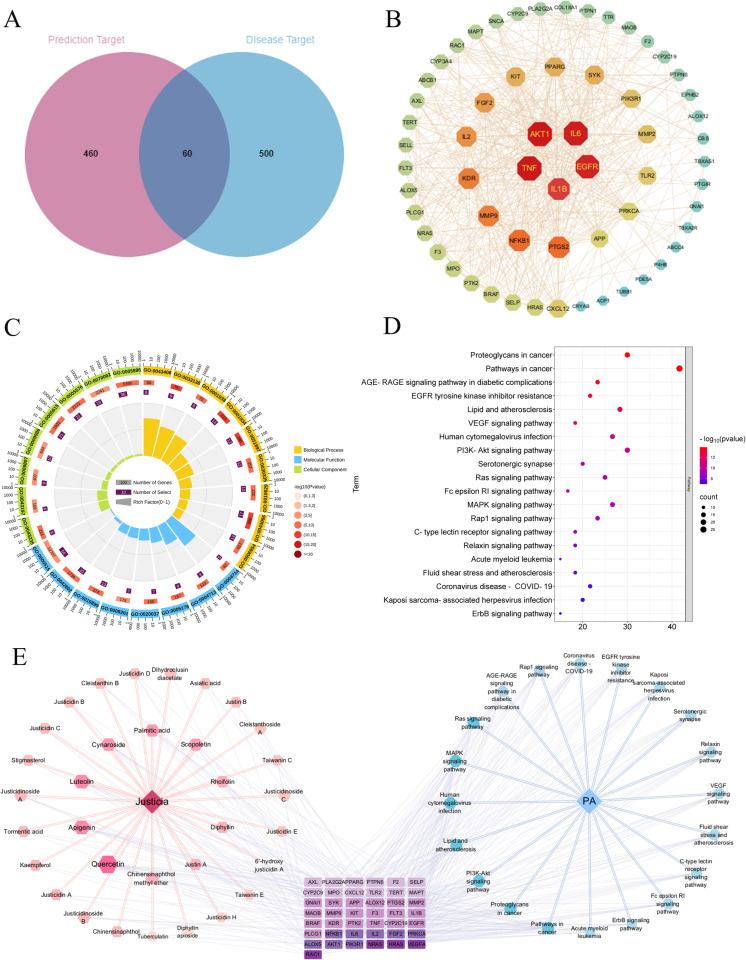
(A) Disease and compound Venn diagram. (B) PPI network of intersection targets. (C) GO functional enrichment analysis of intersection targets. (D) KEGG enrichment analysis of intersection targets. (E) D-C-T-P network diagram of intersection targets. The abbreviations that appear in the pictures are presented in S3, S4, and S5 of the Supporting Information.

Thirty-nine active compounds from the HitPick, SEA, and Target Hunter of Small Molecule Databases were screened, resulting in 235 potential targets. The top 500 disease targets related to platelet aggregation were identified using the Genecards database. The intersection of these two datasets yielded 60 component-disease common targets, and a Venn diagram of these targets was used for visualization (Fig 1A).

The common targets were analyzed using the STRING protein interaction database to construct a PPI network. This network was then reconstructed using Cytoscape 3.10.1 software (Fig 1B). Nodes with larger sizes indicate more critical roles within the network. Key nodes were identified using the CytoNCA plugin based on degree centrality (DC). The top 20 nodes by degree (degree > 15) were RAC-alpha serine/threonine-protein kinase (AKT1), Tumor necrosis factor (TNF), Interleukin-6 (IL6), Epidermal growth factor receptor (EGFR), Interleukin-1 Beta (IL1B), Prostaglandin-endoperoxide synthase 2 (PTGS2), Nuclear factor kappa B1 (NFKB1), Matrix metalloproteinase 9 (MMP9), Kinase insert domain receptor (KDR), Interleukin-2 (IL2), Fibroblast growth factor 2 (FGF2), Spleen tyrosine kinase (SYK), Peroxisome proliferator-activated receptor gamma (PPARG), Stem cell factor receptor (KIT), Phosphoinositide-3-kinase regulatory subunit 1 (PIK3R1), Matrix metalloproteinase 2 (MMP2), CXC chemokine ligand 12 (CXCL12), Toll-like receptor 2 (TLR2), Amyloid precursor protein (APP), and Protein kinase C alpha (PRKCA). This suggests that proteins may be crucial targets for the effective components of *J. procumbens* in combating platelet aggregation-related diseases. The relevant content of proteins and their abbreviations ranked by degree value in PPI network is presented in S1 Table of the supporting information.

The OmicShare tool was used for GO functional enrichment analysis. The results are shown in Fig 1C. The main BPs related to platelet aggregation included positive regulation of protein phosphorylation, negative regulation of apoptotic processes, and positive regulation of protein kinase B signaling. The main CCs were plasma membrane, extracellular space, and extracellular region. MF analysis showed that the targets were mainly associated with enzyme binding, protein tyrosine kinase activity, and integrin binding. Multiple signaling pathways related to cancer, immunity, and inflammation were identified in KEGG enrichment analysis (Fig 1D). Pathways such as EGFR tyrosine kinase inhibitor resistance, lipid and atherosclerosis, the vascular endothelial growth factor (VEGF) signaling pathway, and the PI3K-Akt signaling pathway were closely related to the mechanism of action of *J. procumbens* active compounds in platelet aggregation-related diseases. The relevant content of terminology related to the constituents of the GO enrichment circle diagram is presented in S2 Table of the supporting information.

A D-P-T-C network was constructed, as shown in Fig 1E. The network consisted of 98 nodes (33 compounds, 20 signaling pathways, and 43 targets) and 475 edges. Screening based on degree values (degree > 15) indicated that Ras-related C3 botulinum toxin substrate 1 (RAC1), GTPase NRas (NRAS), GTPase HRas (HRAS), and VEGFA are involved in multiple critical signaling pathways. The key pathways in the network included EGFR tyrosine kinase inhibitor resistance and lipid and atherosclerosis. The analysis results showed that the top 20 compounds in terms of degree value were quercetin, apigenin, luteolin, cynaroside, palmitic acid, scopoletin, chinensinaphthol methyl ether, diphyllin, kaempferol, chinensinaphthol, justicidin A, tormantic acid, justicidinoside B, justicidinoside A, dihydroclusin diacetate, justin B, stigmasterol, justicidin B, asiatic acid, cleistanthin B, justicidin D, and justicidin C. Among them, chinensinaphthol methyl ether, justicidin B, justicidin D, and justicidin C are characteristic components of *J. procumbens* showing potential for treating platelet aggregation-related diseases. The relevant content of proteins and their abbreviations ranked by degree value in D-P-T-C is presented in S3 Table of the supporting information.

### Analysis results of blood components after gavage with the ethyl acetate extract from *J. procumbens*

The preliminary laboratory testing showed the IC_50_ value of the ethyl acetate extract was significantly higher than that of the petroleum ether extract, the n-butanol extract, and the aqueous extract against human platelet aggregation caused by the five inducers (ADP, AA, 5-HT, PAF, and thrombin). Therefore, we conducted an analysis of the composition and bioavailability of the compounds in the ethyl acetate extract*.*

### Chemical composition analysis of ethyl acetate extract

**Fig 2:**
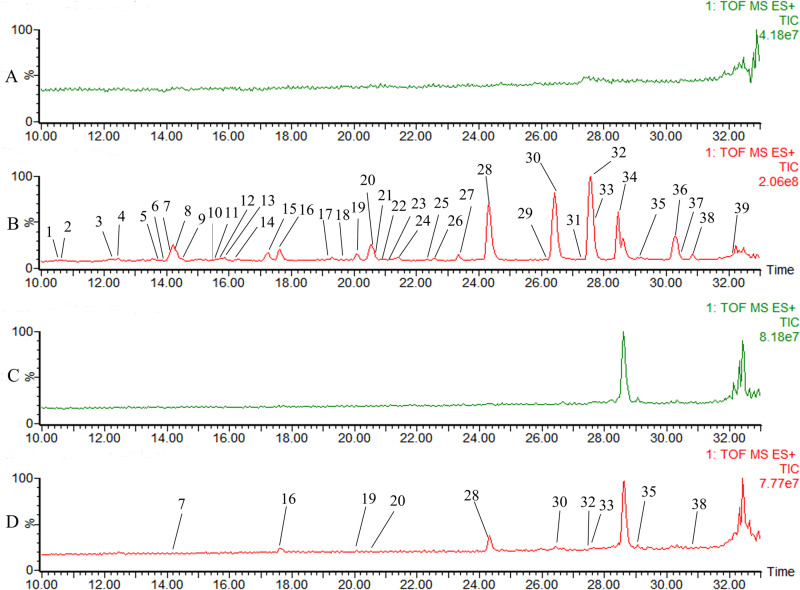
UPLC-MS of the chemical composition and plasma components of ethyl acetate extract from *J. procumbens*. **(A**) Methanol. **(B)** Ethyl acetate extract. **(C)** Blank plasma. **(D)** Ethyl acetate extract components in plasma.

The analysis of the mass spectrometry data revealed 96 components, among which 39 compounds exhibited antithrombotic activity. These compounds primarily belonged to structural types such as 1-arylnaphthalene lignans, 4-arylnaphthalene lignans, dibenzylbutane lignan, and their glycosides. Some structural isomers were identified based on their chromatographic behavior and standard compounds. The amount of 1-arylnaphthalene lignans in *J. procumbens* exceeded that of 4-arylnaphthalene lignans. The glycosyl groups in glycosides contain one to four furanose and pyranose groups. Two sugars compose straight-chain glycosyl, and three or four sugars compose branched-chain glycosyl. Many dibenzylbutane lignans were found in mono- and di-acetylated forms, with a small amount of acetylated sugar residues observed in 1-arylnaphthalene lignan glycosides. The chemical constituents of the ethyl acetate extract of *J. procumbens* are shown in Fig 2B, and the identified compounds are shown in Table 1. The total ion chromatogram of ethyl acetate extract shows that the content of JB, CME, and NJB in arylnaphthalene lignans and the content of 5-methoxy-4,4’-di-O-methylsecolariciresinol diacetate and secoisolariciresinol dimethyl ether diacetate in dibenzylbutane lignans are relatively high.

**Table 1:** Characterization of 39 compounds in the ethyl acetate extract of *J. procumbens.*

	RT(min)	Formula	Parent ion	Exact molecular weight	theoreticalmolecular weight	Identification
1	10.606	C_29_H_42_O_12_	[M+Na]^+^	605.2564	605.2574	Glycoside of 5-methoxy-4,4’-di-O-methylsecolariciresinol
2	10.674	C_28_H_40_O_11_	[M+Na]^+^	575.2479	575.2468	Glycoside of Secoisolariciresinol dimethyl ether
3	12.381	C_32_H_34_O_16_	[M+Na]^+^	697.1721	697.1745	Procumbenoside B
4	12.468	C_27_H_26_O_12_	[M+Na]^+^	565.1340	565.1322	Cleistanthin B
5	13.777	C_27_H_26_O_12_	[M+Na]^+^	565.1340	565.1322	Justicidinoside C
6	13.966	C_32_H_34_O_16_	[M+Na]^+^	697.1721	697.1745	4-O-β-D-apiofuranosyl-(1“‘→6”)-β-D-glucopyranosyldiphyllin
7	14.175	C_23_H_32_O_7_	[M+Na]^+^	443.2025	443.2046	5-methoxy-4,4’-di-O-methylsecolariciresinol
8	14.330	C_22_H_30_O_6_	[M+Na]^+^	413.1956	413.1940	Secoisolariciresinol dimethyl ether
9	14.519	C_28_H_28_O_13_	[M+Na]^+^	595.1405	595.1428	Justicidinoside A
10	15.518	C_41_H_48_O_23_	[M+Na]^+^	931.2506	931.2484	Ciliatoside B
11	15.605	C_31_H_32_O_15_	[M+Na]^+^	667.1660	667.1639	Procumbenoside H
12	15.794	C_28_H_28_O_13_	[M+Na]^+^	595.1405	595.1428	Justicidinoside B
13	15.882	C_31_H_32_O_15_	[M+Na]^+^	667.1660	667.1639	Procumbenoside A
14	16.293	C_36_H_40_O_19_	[M+Na]^+^	799.2054	799.2061	Ciliatoside A
15	17.278	C_26_H_24_O_11_	[M+Na]^+^	535.1223	535.1216	Tuberculatin
16	17.622	C_21_H_16_O_7_	[M+Na]^+^	403.0806	403.0794	Diphyllin
17	19.295	C_20_H_16_O_7_	[M+Na]^+^	391.0812	391.0794	Procumbiene/Juspurpudin
18	19.761	C_21_H_16_O_7_	[M+Na]^+^	403.0806	403.0794	Chinensinaphthol
19	20.105	C_22_H_18_H_8_	[M+Na]^+^	433.0905	433.0899	6‘-hydroxy Justicidin C
20	20.536	C_25_H_34_O_8_	[M+Na]^+^	485.2142	485.2151	5-methoxy-4,4’-di-O-methylsecolariciresinol-9’-monoacetate
21	20.638	C_24_H_32_O_7_	[M+Na]^+^	455.2063	455.2046	Secoisolariciresinol dimethyl ether monoacetate
22	20.914	C_21_H_16_O_7_	[M+Na]^+^	403.0806	403.0794	4’-demethyl chinensinaphthol methyl ether
23	21.157	C_28_H_26_O_12_	[M+Na]^+^	577.1339	577.1322	Diphyllin apioside-2-acetate/Diphyllin apioside-3-acetate
24	21.38	C_21_H_16_O_7_	[M+Na]^+^	403.0806	403.0794	6‘-hydroxy Justicidin B
25	22.345	C_22_H_18_O_8_	[M+Na]^+^	433.0905	433.0899	6‘-hydroxy Justicidin A
26	22.587	C_28_H_26_O_12_	[M+Na]^+^	577.1339	577.1322	Diphyllin apioside-5-acetate
27	23.363	C_26_H_34_O_9_	[M+Na]^+^	513.2089	513.2101	Justin C
28	24.294	C_21_H_16_O_6_	[M+Na]^+^	387.0855	387.0845	Justicidin B
29	26.069	C_23_H_22_O_7_	[M+Na]^+^	433.1245	433.1263	Cilinaphthalide B
30	26.433	C_22_H_18_O_7_	[M+H]^+^	395.1137	395.1131	Chinensinaphthol methyl ether
31	27.276	C_24_H_28_O_8_	[M+Na]^+^	467.1671	467.1682	Justin A
32	27.586	C_27_H_36_O_9_	[M+Na]^+^	527.2281	527.2257	5-methoxy-4,4’-di-O-methylsecolariciresinol diacetate
33	27.674	C_26_H_34_O_8_	[M+Na]^+^	497.2181	497.2151	Secoisolariciresinol dimethyl ether diacetate
34	28.450	C_22_H_18_O_7_	[M+H]^+^	395.1137	395.1131	Neojusticin B
35	29.172	C_21_H_14_O_7_	[M+Na]^+^	401.0620	401.0637	Neojusticin A
36	30.258	C_26_H_32_O_9_	[M+Na]^+^	511.1944	511.1944	(-)-dihydroclusin diacetate
37	30.345	C_25_H_30_O_8_	[M+Na]^+^	481.1841	481.1838	2,3-demethoxysecisolintetralin
38	30.845	C_21_H_14_O_7_	[M+Na]^+^	401.0620	401.0637	Taiwanin E methyl ether
39	32.086	C_24_H_26_O_8_	[M+Na]^+^	465.1525	465.1525	Arirnsin diacetate

### Analysis of constituents migrating into blood after gavage with ethyl acetate extract

Blood samples were collected 1.5 hours after orally administering ethyl acetate extract to SD rats to analyze the constituents that migrated into the blood (Fig 2B, 2C, and 2D). Among these 10 compounds that migrated into the blood, no lignan glycosides were found, indicating that the polarity and molecular weight increase associated with the glycosylation of lignans may hinder their passage through gastrointestinal membranes. No other dibenzylbutane lignan components were detected in the bloodstream, aside from the high content of 5-methoxy-4,4’-di-O-methylsecolariciresinol diacetate, secoisolariciresinol dimethyl ether diacetate, and their derivatives. The higher absorption of arylnaphthalene lignans may be related to their rigid structure. Comparing the molecular ion peak areas of the compounds in plasma and their corresponding compounds in ethyl acetate extract, showed that JB and CME were more easily absorbed through the gastrointestinal tract than other arylnaphthalene lignans. Moreover, these two compounds showed higher content in the ethyl acetate extract; thus, JB and CME were selected for further study. The chemical composition of *J. procumbens* ethyl acetate extract is shown in Table 1, and the ethyl acetate extract compounds that migrated into the blood are shown in Table 2.

**Table 2:** The constituents of ethyl acetate extract entering the blood.

	RT(min)	Formula	Parent ion	Exact molecular weight	theoreticalmolecular weight	Identification
7	14.175	C_23_H_32_O_7_	[M+Na]^+^	443.2025	443.2046	5-methoxy-4,4’-di-O-methylsecolariciresinol
16	17.622	C_21_H_16_O_7_	[M+Na]^+^	403.0806	403.0794	Diphyllin
19	20.105	C_22_H_18_H_8_	[M+Na]^+^	433.0905	433.0899	6‘-hydroxy Justicidin C
20	20.536	C_25_H_34_O_8_	[M+Na]^+^	485.2142	485.2151	5-methoxy-4,4’-di-O-methylsecolariciresinol-9’-monoacetate
28	24.294	C_21_H_16_O_6_	[M+Na]^+^	387.0855	387.0845	Justicidin B
30	26.433	C_22_H_18_O_7_	[M+H]^+^	395.1137	395.1131	Chinensinaphthol methyl ether
32	27.586	C_27_H_36_O_9_	[M+Na]^+^	527.2234	527.2257	5-methoxy-4,4’-di-O-methylsecolariciresinol diacetate
33	27.674	C_26_H_34_O_8_	[M+Na]^+^	497.2135	497.2151	Secoisolariciresinol dimethyl ether diacetate
35	29.172	C_21_H_14_O_7_	[M+Na]^+^	401.0620	401.0637	Neojusticin A
38	30.845	C_21_H_14_O_7_	[M+Na]^+^	401.0620	401.0637	Taiwanin E methyl ether

### Content determination of *J. procumbens* effective extract

**Fig 3:**
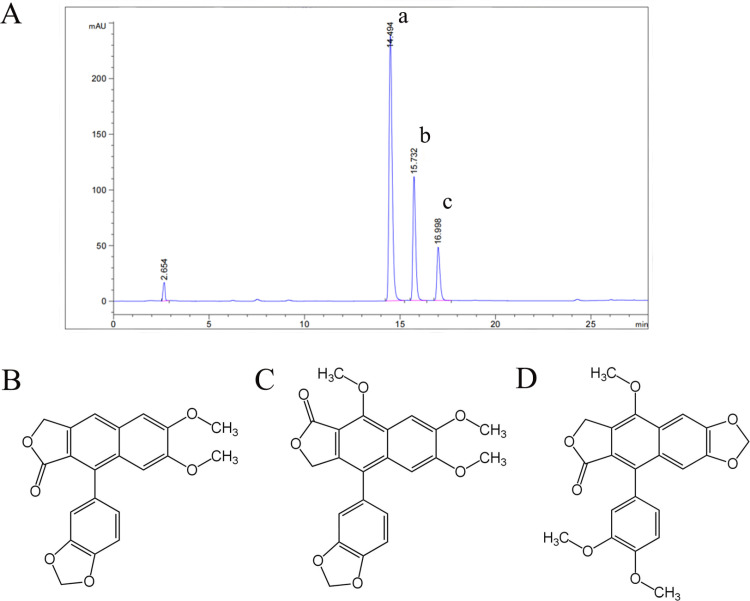
(A) Effective extract chromatogram. (a) JB, (b) CME, and (c) NJB. (B, C, and D) Key compound structures in *J. procumbens* effective extract. (B) Justicidin B., (C) chinensinaphthol methyl ether, and (D) neojusticin B.

JB and CME were used as target compounds for phytochemical separation to prepare the *J. procumbens* effective extract. The content of the major compounds in the effective extract was determined using HPLC and primarily contained JB, CME, and NJB (Fig 3A). The content of each compound is shown in Table 3, and the structure of each compound is shown in Fig 3B-3D. JB and CME play key roles as the effective components of *J. procumbens* against platelet aggregation (NJB was not found in plasma).

**Table 3:** Content of the main compounds in *J. procumbens* effective extract.

Compound	percentage content (%)
JB	45.98
CME	25.06
NJB	19.10

### Results of ethyl acetate extract, effective extract, JB, and CME antiplatelet aggregation tests

This study determined the IC_50_ values of aspirin, JB, CME, ethyl acetate extract, and effective extract against platelet aggregation induced by six agonists, including ADP, PAF, AA, epinephrine, thrombin, and 5-HT. All tested substances exhibited inhibitory effects on platelet aggregation. The effect of CME was stronger than that of JB, and it exhibited a relatively better antiplatelet aggregation effect compared to aspirin. The effective extract showed superior pharmacological effects compared to the ethyl acetate extract, indicating the successful enrichment of antiplatelet aggregation compounds from *J. procumbens* (Table 4). The content of raw platelet aggregation curves and platelet aggregation curve is presented in S1 Fig and S2 Fig of the supporting information.

**Table 4:** IC_50_ of platelet aggregation.

Compound names	IC_50_					
	ADP	Epi	AA	thrombin	5-HT	PAF
Justicidin B	166.16^a^	197.38	45.38^a^	122.75^a^	175.36^a^	60.17^a^
Chinensinaphthol methyl ether	33.82^a^	15.52^a^	30.24^a^	49.20^a^	13.91^a^	20.03^a^
Aspirin	71.53^a^	42.32^a^	20.69^a^	67.36^a^	17.65^a^	40.35^a^
The ethyl acetate extract	0.1315^b^	0.1479^b^	0.0385^b^	0.3625^b^	0.2416^b^	0.3105^b^
The effective extract	0.0827^b^	0.085^b^	0.0196^b^	0.1515^b^	0.1023^b^	0.0370^b^

Note:

^a^: μM;

^b^: mg/mL

### Clot retraction

**Fig 4:**
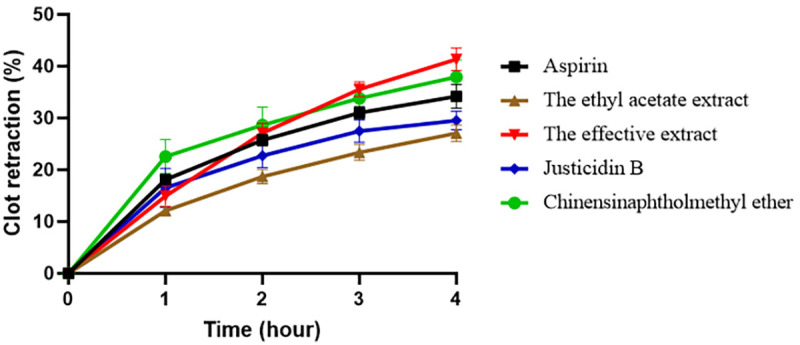
Clot retraction. Note: the black line represents Aspirin, which inhibits clot retraction; the brown line stands for The ethyl acetate extract, showing a weak promotion of clot retraction; the red line indicates The effective extract, strongly promoting clot retraction; the blue line denotes Justicidin B, promoting clot retraction in the early stage but slowing down later; and the green line represents Chinensinaphtholmethyl ether (CME), significantly inhibiting clot retraction.

In the clot retraction experiment, the effects of various drugs on clot retraction were significantly different. The ethyl acetate extract had a weak promoting effect on clot retraction, with relatively low clot retraction rates at each time point, indicating its limited regulation of platelet function. In contrast, the effective extract demonstrated a strong ability to promote clot retraction, with significantly higher clot retraction rates than other drugs, suggesting that its active ingredients positively influenced platelet function and clot structure. JB showed a significant promotion of clot retraction in the early stage, with a rapid increase in the retraction rate, but the growth slowed down in the later stage. Its overall effect was between the above two, reflecting the complexity of its mechanism of action. It might have activated specific platelet mechanisms in the early stage, but was possibly restricted by other factors in the later stage. CME exhibited a significant inhibitory effect on clot retraction, with a retraction rate lower than that of other drugs. It is speculated that it interfered with key platelet functional links and affected the normal clot retraction process (Fig 4).

### Inhibition of FeCl_3_-induced carotid artery thrombosis by the effective extract

#### Observation of blood vessel protection.

**Fig 5:**
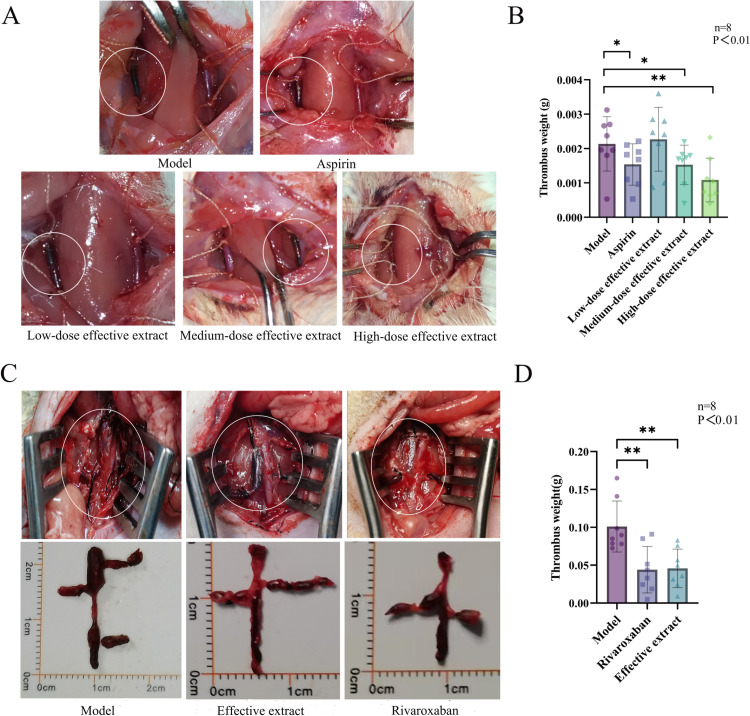
(A) Vascular status during surgery. (B) Weight of thrombi in each group. (C) Thrombosis images from animal experiments in different groups with deep vein thrombosis. (D) Weight of thrombi in each group.

**Fig 6:**
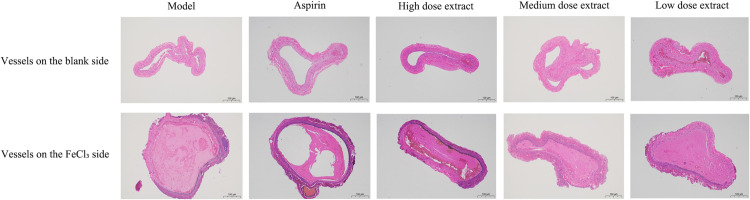
H&E staining results.

After the surgical procedure and removal of the filter paper strip containing FeCl_3_ solution, the blood vessels on the wrapped filter paper side were darkened and thickened, as seen in Fig 5A, in which the inside of the white circle is the wrapped side, and the blood vessels on the other side are the controls. The colors of the blood vessels in the model group, aspirin group, and low-dose extract group were significantly darker than those in the medium and high-dose extract groups, with the latter group having the lightest color. This observation suggests that the high-dose extract group could better protect the blood vessels and significantly reduce damage to the blood vessels from the FeCl_3_ solution. The medium-dose extract group had some protective effects on the blood vessels. The H&E staining results showed that the blood vessels on the modeling side in the model group were completely blocked by thrombi (Fig 6). The aspirin group had the least amount of thrombi. The amount of thrombi gradually increased in the high-dose, medium-dose, and low-dose extract groups. These findings demonstrate that the aspirin group and the high-dose extract group had the best effects of all treatments.

#### Carotid artery thrombosis results.

Measurements of the weight of the thrombi in each group showed that the effective extract had a significant *in vivo* antithrombotic effect and a strong quantitative effect relationship. The medium dose significantly reduced thrombus weight compared with the model group, and the administered dose was only half that of aspirin, with comparable antithrombotic effects. The high dose very significantly reduced thrombus weight, with much higher efficacy than aspirin at only 3/4 of the dose of aspirin. The low-dose group had thrombi weights comparable to those of the model group. The results are shown in Table 5 and Fig 5B.

**Table 5:** Dose and weight of thrombi in each group.

Group	Concentration (mg/ml)	Volume (mL/100 g)	Conversion to adult dose (mg/60 Kg)	Thrombus weight (mg)
Model	0.00	0.75	0.0	2.37 ± 0.53
Aspirin	2.91		218.3	1.68 ± 0.53*
High dose extract	2.02		151.5	1.13 ± 0.74**^#^
Medium dose extract	1.51		113.3	1.69 ± 0.40*
Low dose extract	1.00		74.9	2.47 ± 0.87

* Indicates a significant difference (P < 0.05) compared with the model group; ** indicates a highly significant difference (P < 0.01) compared with the model group; and # indicates a significant difference (P < 0.05) compared with the aspirin group.

### Effective extract proteomics results

**Fig 7:**
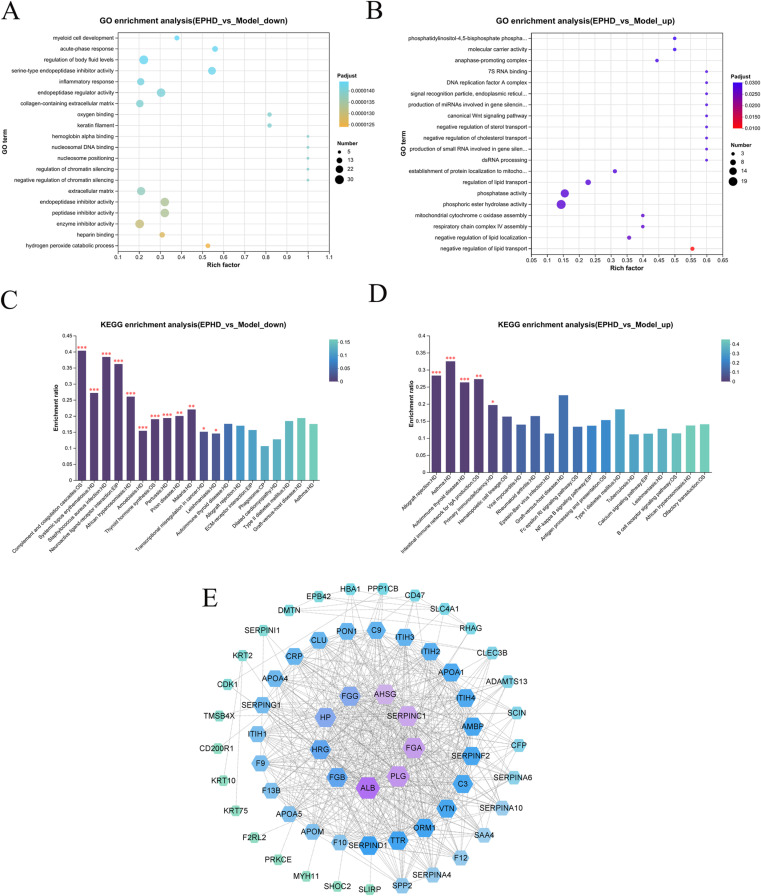
(A) GO analysis of the down-regulated effective extract proteins in proteomics. (B) GO analysis of the up-regulated effective extract proteins in proteomics. (C) KEGG analysis of the down-regulated effective extract proteins in proteomics. (D) KEGG analysis of the up-regulated effective extract proteins in proteomics. (E) PPI diagram of the down-regulated proteins in proteomics. Note: (C) and (D): The abscissa represents the pathway name, and the ordinate represents the enrichment ratio [which is the ratio of the number of proteins enriched in this pathway (protein number) to the number of proteins annotated to the pathway (background number). The larger the ratio, the greater the degree of enrichment]. The color gradient of the columns represents the significance of enrichment. By default, the darker the color, the more significantly enriched the KEGG term. Among them, those with a P or FDR value < 0.001 are marked as ***, those with P or FDR values < 0.01 are marked as **, and those with P or FDR values < 0.05 are marked as *.

The protein mass spectrometry data were uploaded to the Majorbio platform, with parameters set at an up-regulation fold change of 1.2 and a down-regulation fold change of 0.8. Differentially expressed proteins were subjected to GO analysis, with GO annotations and parameters set at an E-value of ≤ 10^-5^, an identity value of ≥ 0.98, and a corrected P-value of ≤ 0.5. A total of 449,876 peaks were detected, of which 104,348 were identified, accounting for 23.19%. The number of identified proteins was 4618. The results of the significant down-regulation of the 20 GO biological processes and the key proteins are shown in Fig 7A. The processes directly related to thrombosis were the acute-phase response and the inflammatory response, among others. The KEGG analysis of these proteins is shown in Fig 7C. The pathways related to thrombophilia included the complement and coagulation cascades signaling pathway and the platelet activation signaling pathway. The core targets included F1LPU1 (proteinase-activated receptor 3, F2rl2), Q5XI31 (GPI transamidase component PIG-S, PIGS), D4A0T9 (ADAM metallopeptidase with thrombospondin type 1 motif, 13, Adamts13), F6Q1N1 (coagulation factor XIII B chain, F13b), Q63207 (coagulation factor X, F10), M0RBF1 (C3-beta-c, C3), P02680 (fibrinogen-gamma chain, Fgg), P16296 (coagulation factor IX, coagulation factor IX, F9), and D3ZTE0 (coagulation factor XII, F12). According to the proteomics results, the targets related to the direct action of the extract on thrombophilia were down-regulated proteins. Those with high relevance were subjected to PPI analysis (Fig 7E).

The extract down-regulated Fgg, Fgb, Fga, F9, F10, and other targets by affecting the complement and coagulation cascades signaling pathway and the platelet activation signaling pathway, thus inhibiting platelet aggregation, reducing the content of coagulation factors and affecting the coagulation pathway, consistent with the multi-database association analysis results. Hence, antithrombosis was affected through both antiplatelet aggregation and anti-coagulation pathways.

The complement and coagulation cascades play important roles in proteomics. As part of the immune system, the complement system is responsible for recognizing and clearing pathogens and damaged cells. Its activation affects the activity of endothelial cells and blood clotting factors, thereby influencing the coagulation process. For instance, complement components such as C3a and C5a can promote inflammatory responses, indirectly affecting coagulation. Conversely, the activation of the coagulation system can also impact the complement system. For example, thrombin can enhance the activation of complement components and increase local inflammation. The formation of deep vein thrombosis (DVT) is closely related to abnormalities in blood coagulation factors, reduced blood flow, and vascular damage. The complement system also plays a crucial role in these processes, as its activation-induced inflammation may increase the risk of thrombosis. Complement components like C5a can damage endothelial cells and exacerbate inflammation, further increasing the tendency for thrombosis. Additionally, interactions between the complement and coagulation systems may amplify coagulation responses, increasing the risk of thrombosis. As the experimental results suggested that the *J. procumbens* effective extract might be effective in treating DVT, we conducted relevant pharmacodynamic tests on animals.

The up-regulation proteomics results showed that processes such as phosphatidylinositol-4,5-bisphosphate phosphatase activity in GO analysis were closely related to the treatment response of cells in thrombotic diseases and involved intracellular signal transduction, lipid metabolism, and enzyme activity regulation (Fig 7B). For example, abnormal lipid metabolism is related to thrombosis. Cells can reduce the risk of thrombosis by negatively regulating sterol and lipid transport and regulating the lipid transport process. Changes in phosphatase and phosphoric ester hydrolase activities affect intracellular signal transduction and metabolic regulation and regulate platelet function or the contraction of vascular smooth muscle cells. In KEGG analysis (Fig 7D), the nuclear factor (NF)-kappa B signaling pathway and others provide clues for understanding the treatment response of cells in thrombosis-related situations as it is associated with thrombosis and thrombosis-related inflammatory responses. Cells can control inflammatory responses by regulating this pathway. The Fc epsilon RI signaling pathway may indirectly affect thrombosis-related immune responses. Cells can adjust this pathway to regulate the activity of immune cells. The antigen processing and presentation pathway may also play a role in thrombosis-related immune responses. Immune cells recognize and process thrombosis-related antigens through this pathway and initiate an adaptive immune response, which helps cells adapt and compensate during thrombosis treatment. The calcium signaling pathway is crucial for the contraction and relaxation of vascular smooth muscle cells and may be related to vascular function regulation after thrombosis. Cells can adapt to vascular changes caused by thrombosis by regulating the calcium signaling pathway and, thus, respond to treatment. The GO and KEGG analysis results indicated that cells in thrombotic diseases may activate compensatory or adaptative mechanisms through multiple pathways, including regulating lipid metabolism, enzyme activity, signal transduction pathways, and immune responses. Subsequent studies should further explore these mechanisms to provide more valuable insight into the treatment of thrombotic diseases.

### Results of animal experiments on deep vein thrombosis

The efficacy test results for DVT are shown in Figs 5C and 5D. Both the rivaroxaban group and the effective extract group exhibited significant reductions in thrombus weight compared to the model group, indicating that they exerted significant antithrombotic effects. However, the difference between the effective extract group and the rivaroxaban group was not significant. The effective extract of *J. procumbens* showed inhibitory effects on both arterial and venous thrombosis at the same dose as aspirin, with significant differences observed.

### Molecular docking results

**Fig 8:**
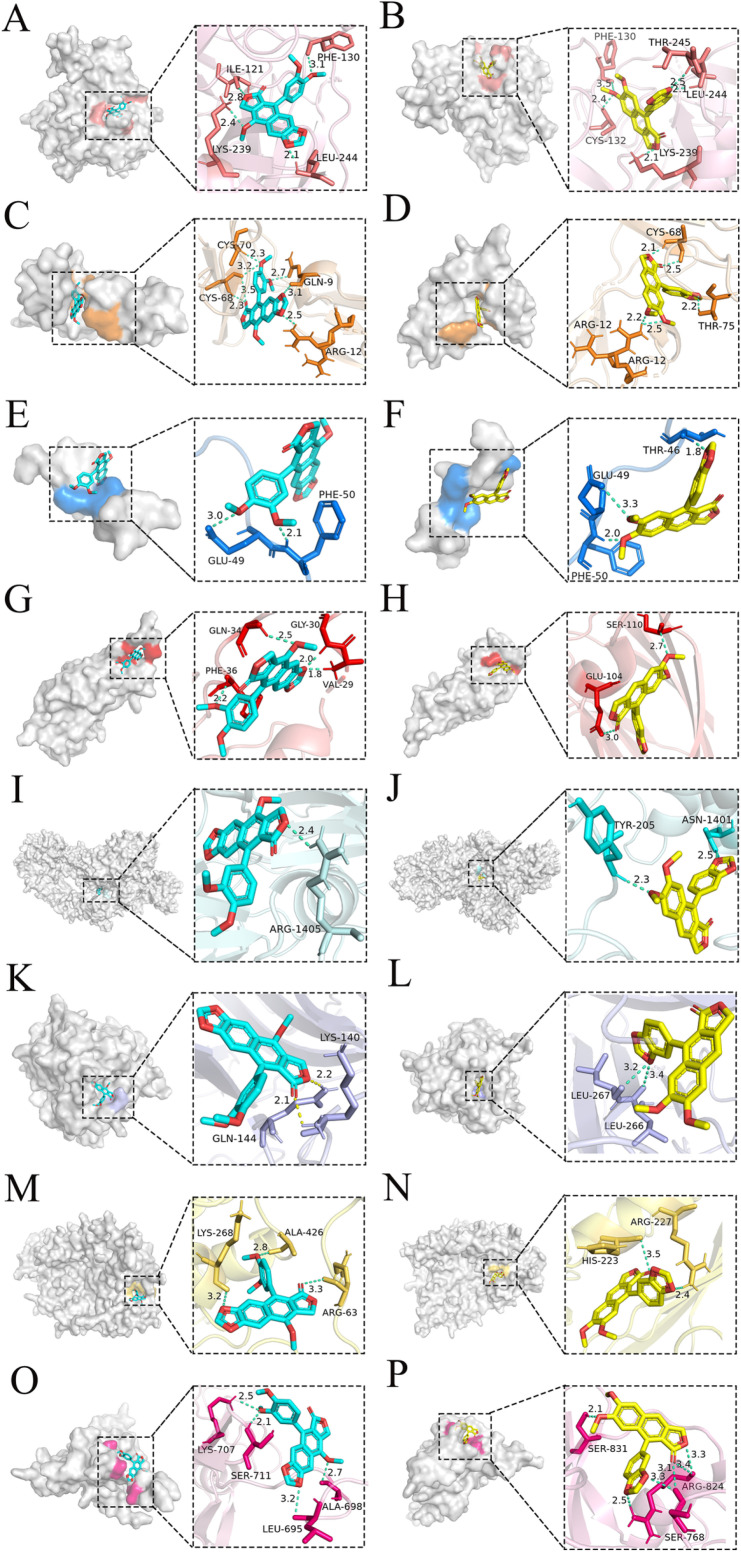
Molecular docking experiment results. **(A)** CME and F9. **(B)** JB and F9. **(C)** CME and F12. **(D)** JB and F12. **(E)** CME and F2rl2. **(F)** JB and F2rl2. **(G)** CME and Prkce. **(H)** JB and Prkce. **(I)** CME and C3. **(J)** JB and C3. **(K)** CME and C1q. **(L)** JB and C1q. **(M)** CME and C8β. **(N)** JB and C8β. **(O)** CME and C7. **(P)** JB and C7.The red color in the small molecule represents oxygen atoms, and the other color parts such as rose, blue, and yellow represent carbon atoms in general.

The virtual binding energies of the compounds in the effective extract with co-regulated proteins were evaluated by molecular docking. The compounds significantly down-regulated in the proteomics results (Prkce, F2rl2, F9, F12, C3, C1q, C8β, and C7) were selected for molecular docking. The PDB database was searched for the preferred human and high-resolution protein structures. 4BDX (PDB ID, coagulation factor XII), 5TNO (PDB ID, coagulation factor IX), 1GMI (PDB ID, protein kinase C ε-type), 2PUX (PDB ID, coagulation factor II thrombin receptor-like 2), 2A73 (PDB ID, complement C3), 5HZF (PDB ID, complement C1q), 3OJY (PDB ID, complement C8β), and 2WCY (PDB ID, complement C7) were selected. Each docking was calculated three times, and the protein-compound docking, including the binding position of the protein to the small molecule, the number and energy of the hydrogen bonds, etc., maintained good stability and consistency. The results can be seen in Fig 8, where different atoms of small molecules are distinguished by different colors. the virtual binding energies of CME with F12, F9, F2rl2, Prkce, C3, C1q, C8β, and C7 were -8.39, -7.52, -5.08, -7.12, -4.19. -4.98, -5.97, and -5.42 kcal/mol, respectively. Those of JB with F12, F9, F2rl2, Prkce, C3, C1q, C8β, and C7 were -8.79, -7.76, -5.72, -6.99, -4.62, -6.84, -6.82, and -5.6 kcal/mol, respectively. After docking of F9 and its corresponding inhibitor (same docking 3 times), the docking binding energies stabilized around -8.26, which are in agreement with the results of related experiments on their strong interaction reported in the literature (APTT and PT tests, etc.), further proving the accuracy of the above docking.

In DVT formation, a significant interplay occurs between complement proteins C3, C1q, C8β, and C7, and coagulation factors F2rl2, F9, and F12. C3, through the degradation products C3a and C3b, enhancing the coagulation process and potentially contributing to thrombus formation. C1q affects the coagulation system by activating the complement cascade and may increase DVT risk through its interaction with immune complexes. C8β and C7 contribute to the formation of the membrane attack complex in the complement system, leading to endothelial cell damage and further increasing the risk of thrombosis. Coagulation factor F2rl2, as a coagulation factor II receptor on platelet membranes, is involved in platelet activation and thrombus formation. Factors F9 (coagulation factor IX) and F12 (coagulation factor XII) are key components of the intrinsic coagulation pathway and directly participate in blood coagulation. Abnormal activation of the complement system may modulate the function of these coagulation factors, influencing thrombus formation. Thus, complement proteins C3, C1q, C8β, and C7 regulate coagulation and thrombosis through their interactions with F2rl2, F9, and F12, highlighting their critical roles in the development of DVT.

JB and CME mainly bind to two endogenous coagulation factors, F12 and F9, which may be involved in the endogenous coagulation pathway and down-regulate endogenous coagulation factors, thereby inhibiting the coagulation process. F2rl2 is a coagulation factor II receptor on platelet membranes, which is involved in the platelet activation process, and Prkce is a protein kinase C epsilon type, which is an important mediator of vascular permeability. It has been associated with a variety of vascular diseases, including endothelial damage and tissue damage associated with smoking. These proteins may be targets of the antithrombotic activity of *J. procumbens* effective extract.

Overall, the molecular docking binding scores of F2rl2 were not low but were significantly down-regulated in proteomics, allowing speculation that the compounds in the effective extract may not down-regulate the expression of F2rl2 by directly binding to the protein. All compounds in the effective extract associated with F12 had the lowest binding energies, suggesting that F12 may be a potential target (Fig 8).

## Discussion

Clinically, antiplatelet agents and anticoagulants inhibit thrombosis, have high efficacy in preventing thrombotic diseases, and can reduce the incidence of acute myocardial infarction and mortality [4]. *J. procumbens* was recorded as having blood-activating effects in the 17th century Chinese material medica Compendium of Materia Medica by Li Shizhen. Since ancient times, plant extracts have been documented around the globe for treating disease, and the pharmacological effects of plant constituents are being increasingly understood. Previous laboratory studies also showed that the extract of *J. procumbens* has antiplatelet aggregation effects [20–21]. This study investigated the feasibility of developing *J. procumbens* as a functional food for consumption by patients with thrombosis.

First, a multi-database correlation analysis of *J. procumbens* was performed to predict antiplatelet aggregation function. The analysis showed that the antithrombotic effect might be achieved by modulating the platelet activation pathway and the PI3K-Akt signaling pathway. After confirming the antiplatelet potential of *J. procumbens*, we used UPLC-MS to analyze the chemical composition and compounds migrating into the blood of rats treated with the ethyl acetate extract of this plant. Based on serum medicinal chemistry results, we used percolation extraction and silica gel column chromatography to prepare *J. procumbens* effective extract and analyzed the chemical constituents using HPLC. The preparation method is simple; without the need for special reagents, the production cost is low, and the quality of the prepared extracts can be controlled. Through *in vitro* experiments on antiplatelet aggregation and clot retraction, we found that the ethyl acetate extract, the effective extract, JB, and CME all exhibited antiplatelet aggregation effects. An FeCl_3_-induced carotid artery thrombosis model in SD rats showed that high-dose extract could effectively protect the blood vessels and significantly reduce FeCl_3_-induced damage. The pharmacological efficacy of the effective extract was superior to that of aspirin. SD rat platelets were isolated for proteomics studies to elucidate the antithrombotic mechanism of the effective extract. The extract was found to be effective in protecting blood vessels by affecting the complement and coagulation cascades signaling pathway and the platelet activation signaling pathway. Animal experiments on DVT showed that the effective parts of *J. procumbens* also exert certain anti-venous thrombus effects. At the same dosage as aspirin, *J. procumbens* was observed to inhibit both arterial and venous thrombi, with significant differences. The results of molecular docking showed that Prkce, F2rl2, F12, F9, C3, C1q, C8β, and C7 may be targets of JB and CME. In previous research [22–23], integrin αIIbβ3 was shown to mediate platelet interaction by binding to ligands such as fibrinogen. PKCα, PI3Kγ, and MAPK14 participate in different signaling pathways to regulate platelet activation and aggregation. The Gq-PLC-PKC and Gi-PI3K-MAPK signaling pathways provide an important framework for understanding this mechanism. In the current study, the new targets identified may participate in different signaling networks and interact with known targets to regulate platelet activation and coagulation cascades. For example, Prkce may play a key role in a specific signaling pathway, and F2rl2 and F12 may interact with coagulation factors. JB and CME may regulate platelet function by affecting both new and known targets, involving multiple molecular mechanisms.

The expansion of our current study not only enriches our understanding of the mechanism of platelet aggregation inhibition but also provides more potential targets for the development of new antiplatelet aggregation drugs. In the future, we will further study the interaction between the new and known targets, the specific mechanisms of JB and CME, and their synergistic effects with other drugs. We also plan to conduct clinical studies to verify the effectiveness and safety of these new targets.

## Conclusion

In summary, the raw materials of *J. procumbens* are easy to obtain, the process of preparing the extract rich in arylnaphthalene lignans is simple, and it has a strong antiplatelet aggregation effect. *J. procumbens* can exert a certain therapeutic effect on both arterial and venous thrombosis. Therefore, *J. procumbens* shows good prospects and potential in the development of functional health care drugs.

## Supporting information

S1 File Details of proteomics search parameters. (DOC)

S1 Table Proteins and their abbreviations ranked by degree value in PPI network. (DOC)

S2 Table Terminology related to the constituents of the GO enrichment circle diagram. (DOC)

S3 Table Proteins and their abbreviations ranked by degree value in D-P-T-C. (DOC)

S1 Fig Raw platelet aggregation curves. (DOC)

S2 Fig Platelet aggregation curve. (DOC)
