# Supplementary material for: Antithrombotic effect and plasma pharmacochemistry of Justicia Procumbens L
Source: PLoS One. 2025 Apr 30;20(4):e0321023. doi: 10.1371/journal.pone.0321023 (PMC12083875; doi:10.1371/journal.pone.0321023)
Supplement: S1 File — (DOC) [file pone.0321023.s001.doc]

**S1 File. Details of Proteomics Search Parameters**

The following are the relevant software and version information:
(1) Pfam database, Version 33.1, for full-protein function annotation, website: <http://pfam.xfam.org/>.
(2) KEGG database, Version 2020.07, for full-protein function annotation, website: <http://www.genome.jp/kegg/>.
(3) eggNOG database, Version 2020.06, for full-protein function annotation, website: [http://eggnogdb.embl.de/#/app/home](http://eggnogdb.embl.de/#/app/home).
(4) Swiss-prot database, Version 2020.06, for full-protein function annotation, download address: [ftp://ftp.uniprot.org/pub/databases/uniprot/current_release/knowledgebase/complete/uniprot_sprot.fasta.gz](https://ftp//ftp.uniprot.org/pub/databases/uniprot/current_release/knowledgebase/complete/uniprot_sprot.fasta.gz).
(5) NCBI species classification database, Version 2020.06, for full-protein function annotation, download address: [ftp://ftp.ncbi.nlm.nih.gov/pub/taxonomy/accession2taxid/nucl_gb.accession2taxid.gz](https://ftp//ftp.ncbi.nlm.nih.gov/pub/taxonomy/accession2taxid/nucl_gb.accession2taxid.gz).
(6) GO database, Version 2020.0628, for full-protein function annotation, website: <http://www.geneontology.org/>.
(7) NR database, Version 2020.06, for full-protein function annotation, website: <https://www.ncbi.nlm.nih.gov/public/>.
(8) PIR idmapping database, Version 2020.06, for full-protein function annotation, download address: [ftp://ftp.pir.georgetown.edu/databases/idmapping/idmapping.tb.gz](https://ftp//ftp.pir.georgetown.edu/databases/idmapping/idmapping.tb.gz).
(9) For data quality control, we use self-compiled software by Meiji.
(10) For expression analysis, we use DIAMOND, version v0.8.37.99, for protein function annotation (NR, Swiss-Prot).
(11) For protein function annotation (Pfam), we use HMMER, version 3.1b2.
(12) For protein subcellular localization annotation, we use MultiLoc2.
(13) For full-protein function annotation (GO), we use BLAST2GO, version 2.5.0.
(14) For full-protein function annotation (KEGG), we use KOBAS, version 2.1.1.
(15) For expression difference analysis, we use R.
(16) For protein set analysis (GO enrichment), we use goatools, version 0.6.5.
(17) For protein set analysis (KEGG enrichment), we use Python.
(18) For Mfuzz time series analysis, we use Mfuzz, Version 2.6.0, download address: <https://www.bioconductor.org/packages/release/bioc/html/Mfuzz.html>.
In this study, we control the false discovery rate (FDR) to be less than 0.01. The peptide mass tolerance and fragment ion tolerance are less than 10 ppm. For protein or peptide identification and quantification, the minimum number of peptides required is greater than or equal to 1.
