# Supplementary material for: Antithrombotic effect and plasma pharmacochemistry of Justicia Procumbens L
Source: PLoS One. 2025 Apr 30;20(4):e0321023. doi: 10.1371/journal.pone.0321023 (PMC12083875; doi:10.1371/journal.pone.0321023)
Supplement: S1 Table — (DOC) [file pone.0321023.s002.doc]

**S1 Table Proteins and Their Abbreviations Ranked by Degree Value in PPI Network**

Table Proteins and Their Abbreviations Ranked by Degree Value in PPI Network

| Top | Degree Value | Protein (from Uniprot) | Abbreviation |
| --- | --- | --- | --- |
| 1 | 42.0 | RAC-alpha serine/threonine-protein kinase | AKT1 |
| 2 | 42.0 | Tumor necrosis factor | TNF |
| 3 | 41.0 | Interleukin-6 | IL6 |
| 4 | 40.0 | Epidermal growth factor receptor | EGFR |
| 5 | 35.0 | Interleukin-1 beta | IL1B |
| 6 | 34.0 | Prostaglandin G/H synthase 2 | PTGS2 |
| 7 | 34.0 | Nuclear factor NF-kappa-B p105 subunit | NFKB1 |
| 8 | 32.0 | Matrix metalloproteinase-9 | MMP9 |
| 9 | 32.0 | Vascular endothelial growth factor receptor 2 | KDR |
| 10 | 30.0 | Interleukin-2 | IL2 |
| 11 | 29.0 | Fibroblast growth factor 2 | FGF2 |
| 12 | 27.0 | Tyrosine-protein kinase SYK | SYK |
| 13 | 27.0 | Peroxisome proliferator-activated receptor gamma | PPARG |
| 14 | 27.0 | Mast/stem cell growth factor receptor Kit | KIT |
| 15 | 25.0 | Phosphatidylinositol 3-kinase regulatory subunit alpha | PIK3R1 |
| 16 | 25.0 | 72 kDa type IV collagenase | MMP2 |
| 17 | 24.0 | Stromal cell-derived factor 1 | CXCL12 |
| 18 | 24.0 | Toll-like receptor 2 | TLR2 |
| 19 | 23.0 | Amyloid-beta precursor protein | APP |
| 20 | 23.0 | Protein kinase C alpha type | PRKCA |
| 21 | 21.0 | GTPase HRas | HRAS |
| 22 | 20.0 | P-selectin | SELP |
| 23 | 20.0 | Serine/threonine-protein kinase B-raf | BRAF |
| 24 | 19.0 | Focal adhesion kinase 1 | PTK2 |
| 25 | 19.0 | Tissue factor | F3 |
| 26 | 19.0 | Myeloperoxidase | MPO |
| 27 | 18.0 | GTPase NRas | NRAS |
| 28 | 18.0 | 1-phosphatidylinositol 4,5-bisphosphate phosphodiesterase gamma-1 | PLCG1 |
| 29 | 17.0 | Polyunsaturated fatty acid 5-lipoxygenase | ALOX5 |
| 30 | 16.0 | L-selectin | SELL |
| 31 | 16.0 | Telomerase reverse transcriptase | TERT |
| 32 | 16.0 | Tyrosine-protein kinase receptor UFO | AXL |
| 33 | 16.0 | Receptor-type tyrosine-protein kinase FLT3 | FLT3 |
| 34 | 16.0 | Cytochrome P450 3A4 | CYP3A4 |
| 35 | 16.0 | ATP-dependent translocase ABCB1 | ABCB1 |
| 36 | 15.0 | Ras-related C3 botulinum toxin substrate 1 | RAC1 |
| 37 | 15.0 | Microtubule-associated protein tau | MAPT |
| 38 | 14.0 | Alpha-synuclein | SNCA |
| 39 | 13.0 | Cytochrome P450 2C9 | CYP2C9 |
| 40 | 12.0 | Phospholipase A2, membrane associated | PLA2G2A |
| 41 | 12.0 | Collagen alpha-1(XVIII) chain | COL18A1 |
| 42 | 12.0 | Tyrosine-protein phosphatase non-receptor type 1 | PTPN1 |
| 43 | 11.0 | Transthyretin | TTR |
| 44 | 11.0 | Amine oxidase [flavin-containing] B | MAOB |
| 45 | 10.0 | Prothrombin | F2 |
| 46 | 10.0 | Cytochrome P450 2C19 | CYP2C19 |
| 47 | 9.0 | Tyrosine-protein phosphatase non-receptor type 6 | PTPN6 |
| 48 | 8.0 | Ephrin type-B receptor 2 | EPHB2 |
| 49 | 7.0 | Polyunsaturated fatty acid lipoxygenase ALOX12 | ALOX12 |
| 50 | 7.0 | Cystathionine beta-synthase | CBS |
| 51 | 6.0 | Prostacyclin receptor | PTGIR |
| 52 | 6.0 | Thromboxane-A synthase | TBXAS1 |
| 53 | 5.0 | Guanine nucleotide-binding protein G(i) subunit alpha-1 | GNAI1 |
| 54 | 5.0 | Thromboxane A2 receptor | TBXA2R |
| 55 | 4.0 | Protein disulfide-isomerase | P4HB |
| 56 | 4.0 | ATP-binding cassette sub-family C member 4 | ABCC4 |
| 57 | 3.0 | cGMP-specific 3',5'-cyclic phosphodiesterase | PDE5A |
| 58 | 2.0 | Alpha-crystallin B chain | CRYAB |
| 59 | 2.0 | Tubulin beta-1 chain | TUBB1 |
| 60 | 2.0 | Low molecular weight phosphotyrosine protein phosphatase | ACP1 |
