# Supplementary material for: Antithrombotic effect and plasma pharmacochemistry of Justicia Procumbens L
Source: PLoS One. 2025 Apr 30;20(4):e0321023. doi: 10.1371/journal.pone.0321023 (PMC12083875; doi:10.1371/journal.pone.0321023)
Supplement: S2 Table — (DOC) [file pone.0321023.s003.doc]

**S2 Table Terminology Related to the Constituents of the GO Enrichment Circle Diagram (each category is sorted by P-value from smallest to largest)**

**Table** Terminology Related to the Constituents of the GO Enrichment Circle Diagram (each category is sorted by P-value from smallest to largest)

| Catalogue | GO ID | GO Term |
| --- | --- | --- |
| BP | GO:0001934 | positive regulation of protein phosphorylation |
|  | GO:0043066 | negative regulation of apoptotic process |
|  | GO:0051897 | positive regulation of protein kinase B signaling |
|  | GO:0033138 | positive regulation of peptidyl-serine phosphorylation |
|  | GO:0030335 | positive regulation of cell migration |
|  | GO:0043406 | positive regulation of MAP kinase activity |
|  | GO:0001938 | positive regulation of endothelial cell proliferation |
|  | GO:0010629 | negative regulation of gene expression |
|  | GO:0006954 | inflammatory response |
| CC | GO:0005886  GO:0005615  GO:0005576  GO:0043235  GO:0009897  GO:0070062 | plasma membrane  extracellular space  extracellular region  receptor complex  external side of plasma membrane  extracellular exosome |
|  | GO:0009986  GO:0043197 | cell surface  dendritic spine |
|  | GO:0019899 | enzyme binding |
|  | GO:0004713 | protein tyrosine kinase activity |
|  | GO:0005178 | integrin binding |
|  | GO:0042802 | identical protein binding |
| MF | GO:0020037 | heme binding |
|  | GO:0004714 | transmembrane receptor protein tyrosine kinase activity |
|  | GO:0008201 | heparin binding |
|  | GO:0005515 | Protein binding |
