# Supplementary material for: Antithrombotic effect and plasma pharmacochemistry of Justicia Procumbens L
Source: PLoS One. 2025 Apr 30;20(4):e0321023. doi: 10.1371/journal.pone.0321023 (PMC12083875; doi:10.1371/journal.pone.0321023)
Supplement: S3 Table — (DOC) [file pone.0321023.s004.doc]

**S3 Table Proteins and Their Abbreviations Ranked by Degree Value in D-P-T-C**

**Table**

Proteins and Their Abbreviations Ranked by Degree Value in D-P-T-C

| Top | Degree Value | Protein (from Uniprot) | Abbreviation |
| --- | --- | --- | --- |
| 1 | 26.0 | Ras-related C3 botulinum toxin substrate 1 | RAC1 |
| 2 | 20.0 | Vascular endothelial growth factor A, long form | VEGFA |
| 3 | 20.0 | GTPase HRas | HRAS |
| 4 | 20.0 | GTPase NRas | NRAS |
| 5 | 19.0 | Phosphatidylinositol 3-kinase regulatory subunit alpha | PIK3R1 |
| 6 | 19.0 | RAC-alpha serine/threonine-protein kinase | AKT1 |
| 7 | 19.0 | Polyunsaturated fatty acid 5-lipoxygenase | ALOX5 |
| 8 | 17.0 | Protein kinase C alpha type | PRKCA |
| 9 | 16.0 | Fibroblast growth factor 2 | FGF2 |
| 10 | 16.0 | Interleukin-2 | IL2 |
| 11 | 16.0 | Interleukin-6 | IL6 |
| 12 | 15.0 | Nuclear factor NF-kappa-B p105 subunit | NFKB1 |
| 13 | 14.0 | 1-phosphatidylinositol 4,5-bisphosphate phosphodiesterase gamma-1 | PLCG1 |
| 14 | 12.0 | Epidermal growth factor receptor | EGFR |
| 15 | 11.0 | Cytochrome P450 2C19 | CYP2C19 |
| 16 | 10.0 | Tumor necrosis factor | TNF |
| 17 | 9.0 | Focal adhesion kinase 1 | PTK2 |
| 18 | 9.0 | Vascular endothelial growth factor receptor 2 | KDR |
| 19 | 9.0 | Serine/threonine-protein kinase B-raf | BRAF |
| 20 | 8.0 | Interleukin-1 beta | IL1B |
| 21 | 8.0 | Receptor-type tyrosine-protein kinase FLT3 | FLT3 |
| 22 | 8.0 | Tissue factor | F3 |
| 23 | 7.0 | Mast/stem cell growth factor receptor Kit | KIT |
| 24 | 7.0 | Matrix metalloproteinase-9 | MMP9 |
| 25 | 7.0 | Amine oxidase [flavin-containing] B | MAOB |
| 26 | 7.0 | 72 kDa type IV collagenase | MMP2 |
| 27 | 7.0 | Prostaglandin G/H synthase 2 | PTGS2 |
| 28 | 6.0 | Polyunsaturated fatty acid lipoxygenase ALOX12 | ALOX12 |
| 29 | 6.0 | Amyloid-beta precursor protein | APP |
| 30 | 6.0 | Tyrosine-protein kinase SYK | SYK |
| 31 | 6.0 | Guanine nucleotide-binding protein G(i) subunit alpha-1 | GNAI1 |
| 32 | 5.0 | Microtubule-associated protein tau | MAPT |
| 33 | 5.0 | Telomerase reverse transcriptase | TERT |
| 34 | 5.0 | Toll-like receptor 2 | TLR2 |
| 35 | 4.0 | Stromal cell-derived factor 1 | CXCL12 |
| 36 | 4.0 | Myeloperoxidase | MPO |
| 37 | 3.0 | Cytochrome P450 2C9 | CYP2C9 |
| 38 | 3.0 | P-selectin | SELP |
| 39 | 3.0 | Prothrombin | F2 |
| 40 | 3.0 | Tyrosine-protein phosphatase non-receptor type 6 | PTPN6 |
| 41 | 3.0 | Peroxisome proliferator-activated receptor gamma | PPARG |
| 42 | 2.0 | Phospholipase A2, membrane associated | PLA2G2A |
| 43 | 2.0 | Tyrosine-protein kinase receptor UFO | AXL |
