# Supplementary material for: Antithrombotic effect and plasma pharmacochemistry of Justicia Procumbens L
Source: PLoS One. 2025 Apr 30;20(4):e0321023. doi: 10.1371/journal.pone.0321023 (PMC12083875; doi:10.1371/journal.pone.0321023)
Supplement: S1 Fig — (DOC) [file pone.0321023.s005.doc]

**S1 Fig. Raw Platelet Aggregation Curves**

**Blank (5-HT)**

**
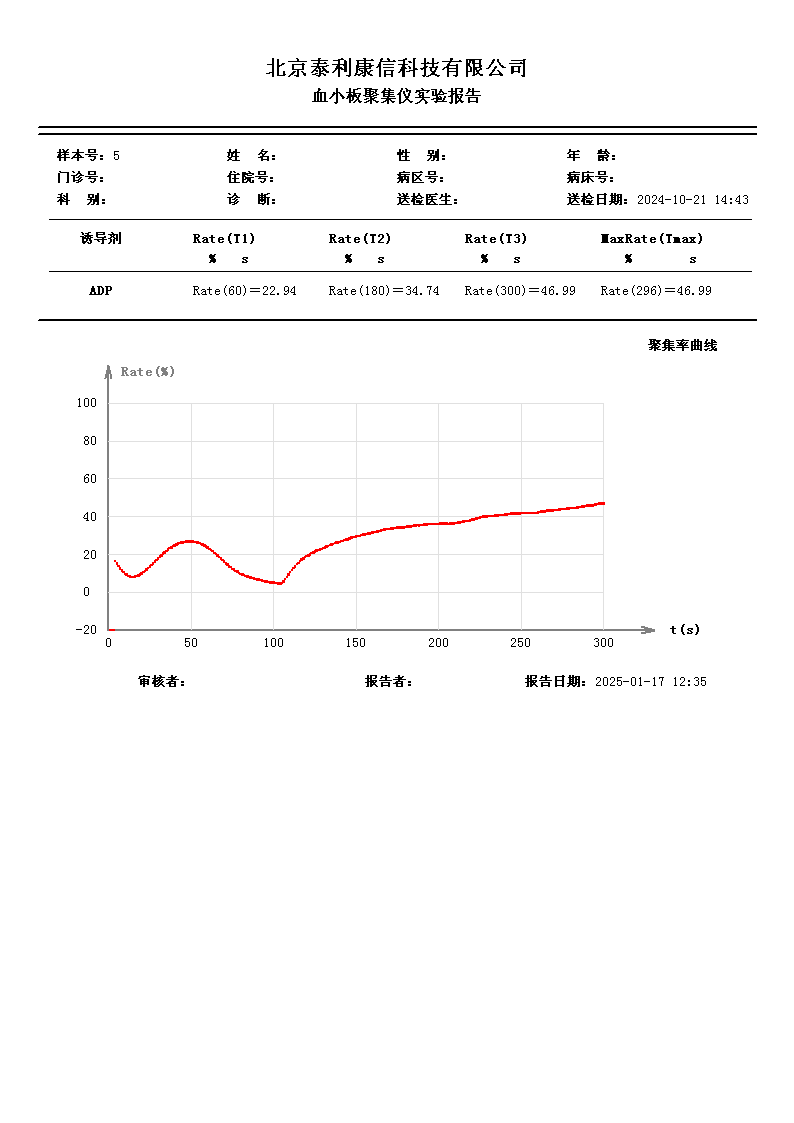
**

**The effective extract (5-HT)**

**
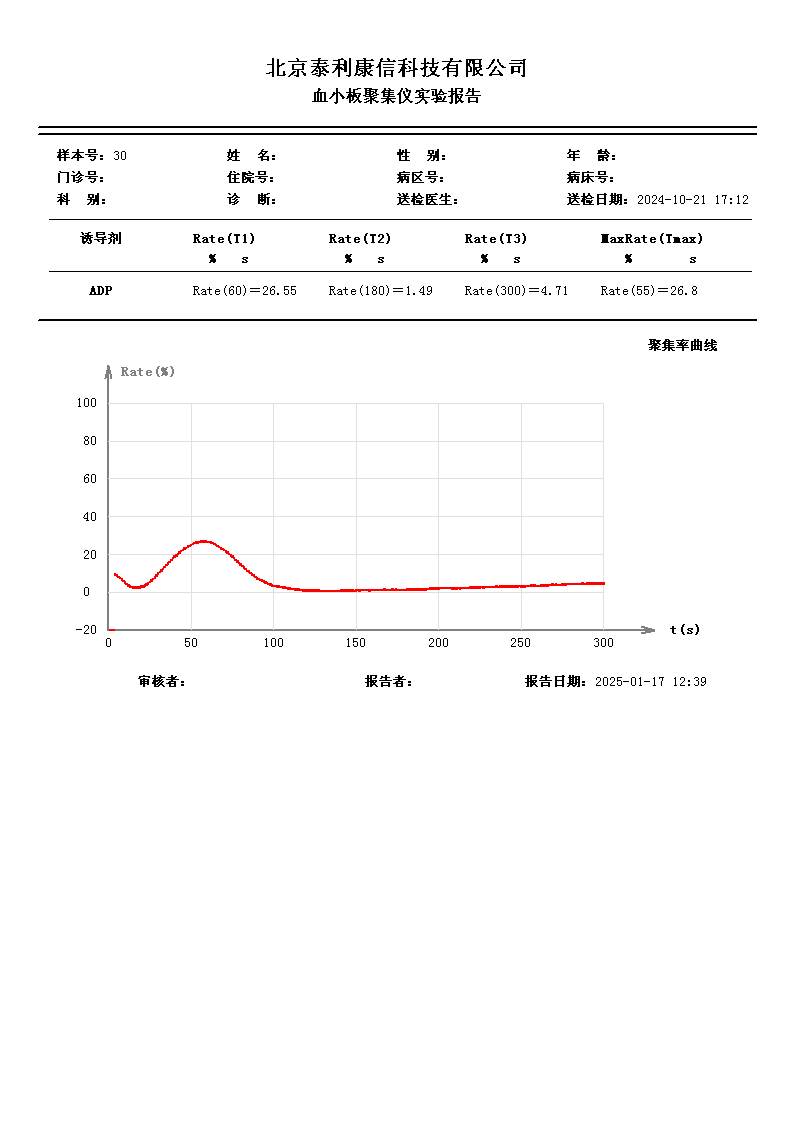
**

**Blank (AA)**

**
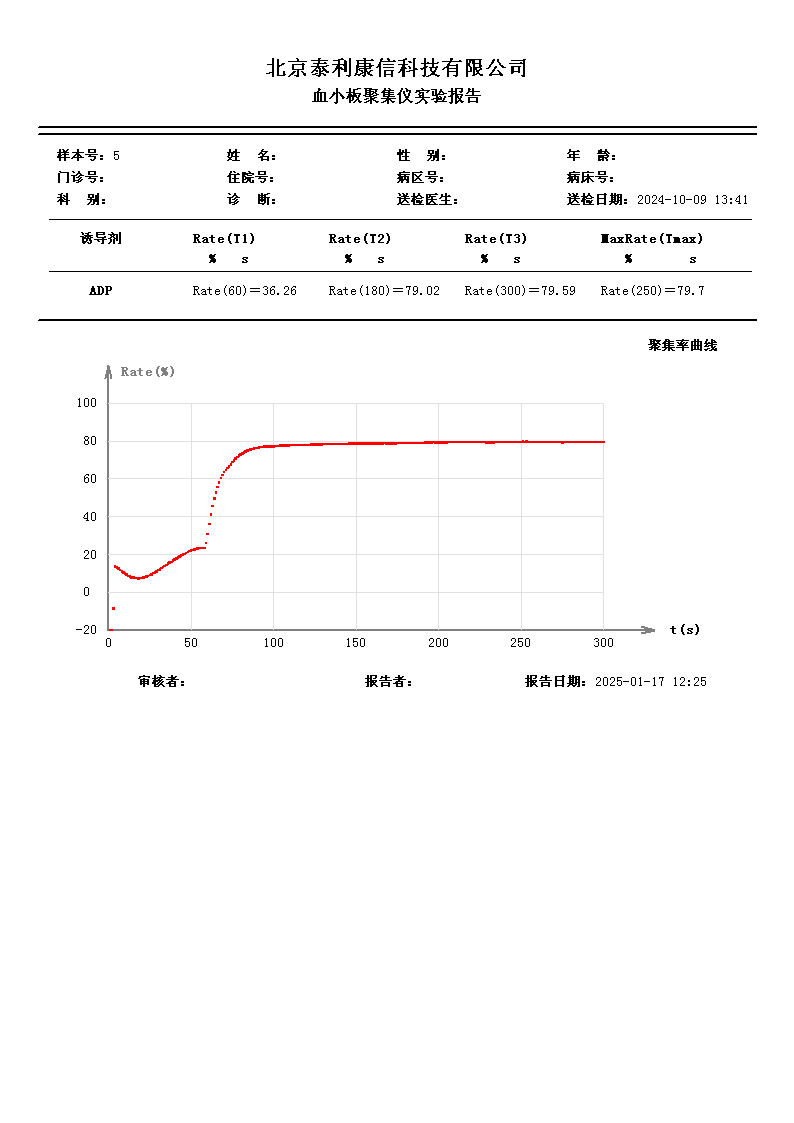
**

**The effective extract (AA)**

**
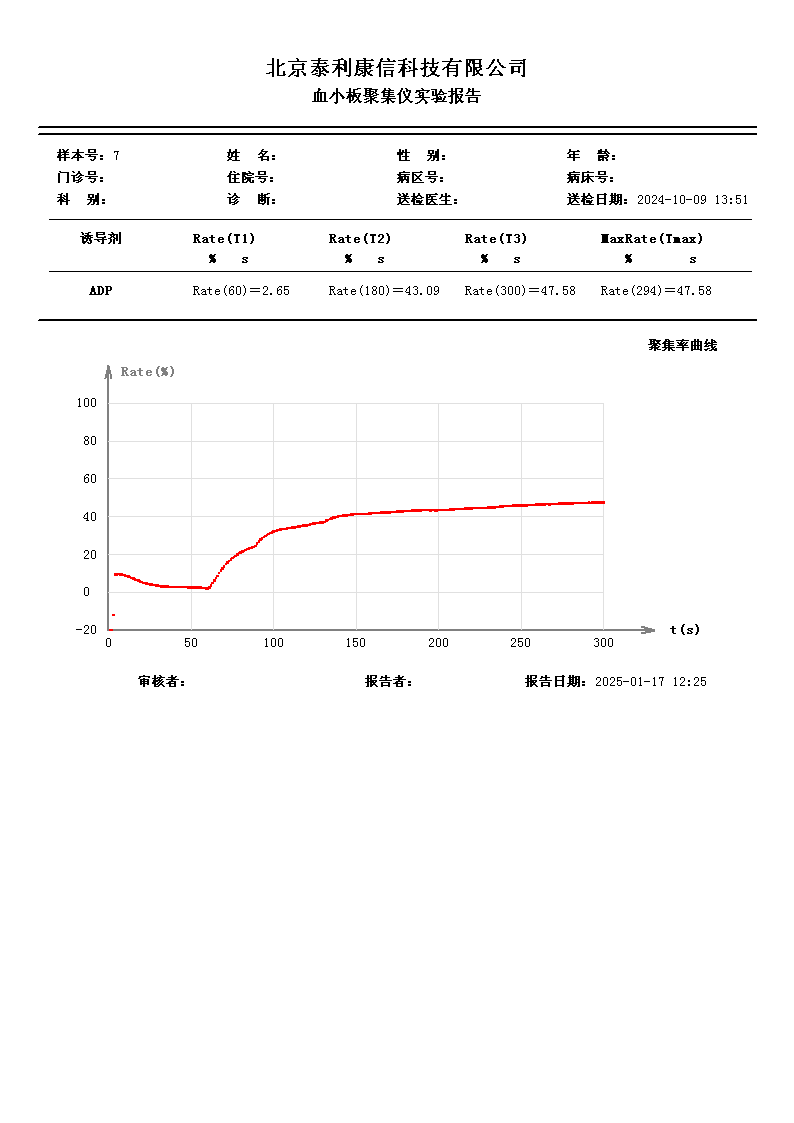
**

**Blank (ADP)**

**
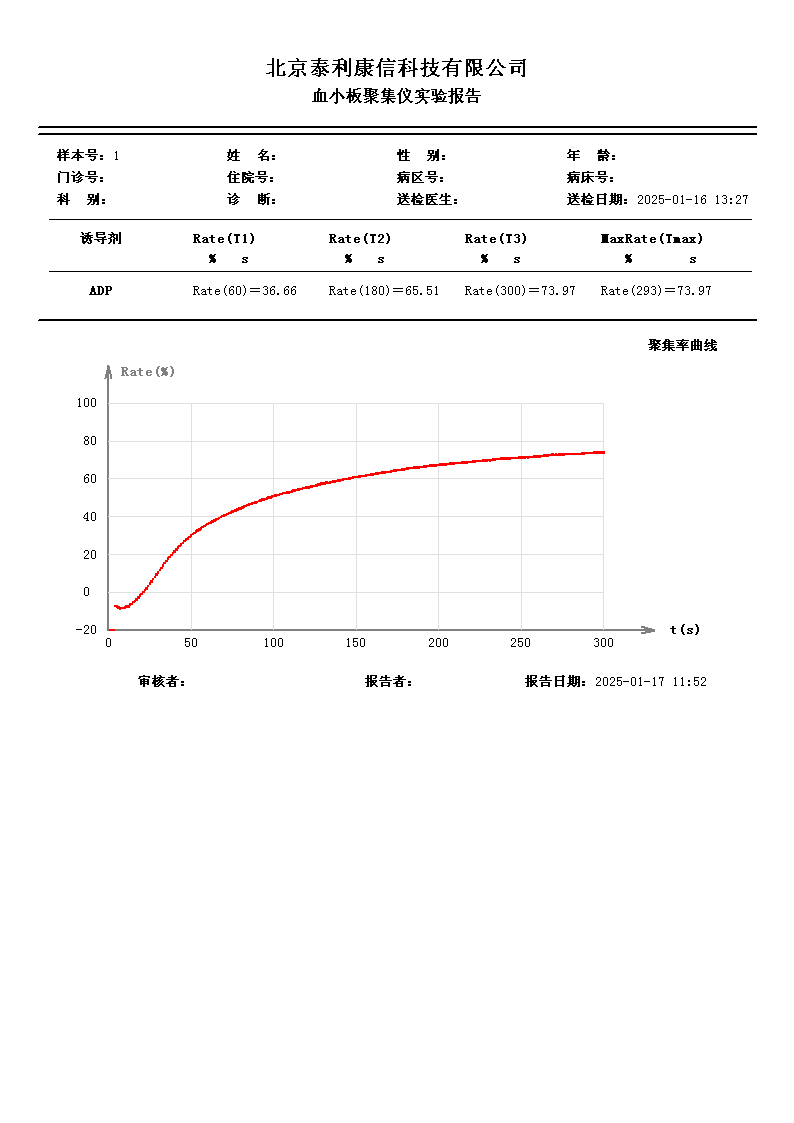
**

**The effective extract (ADP)**

**
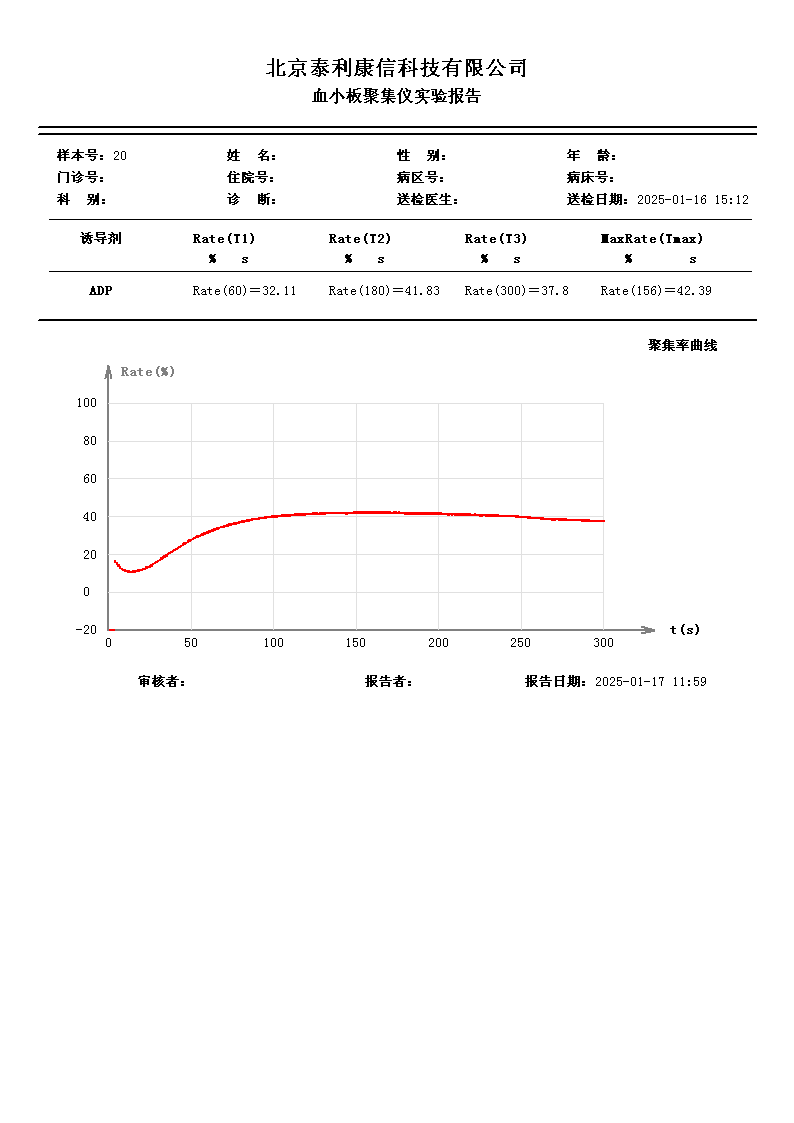
**

**Blank (EPI)**

**
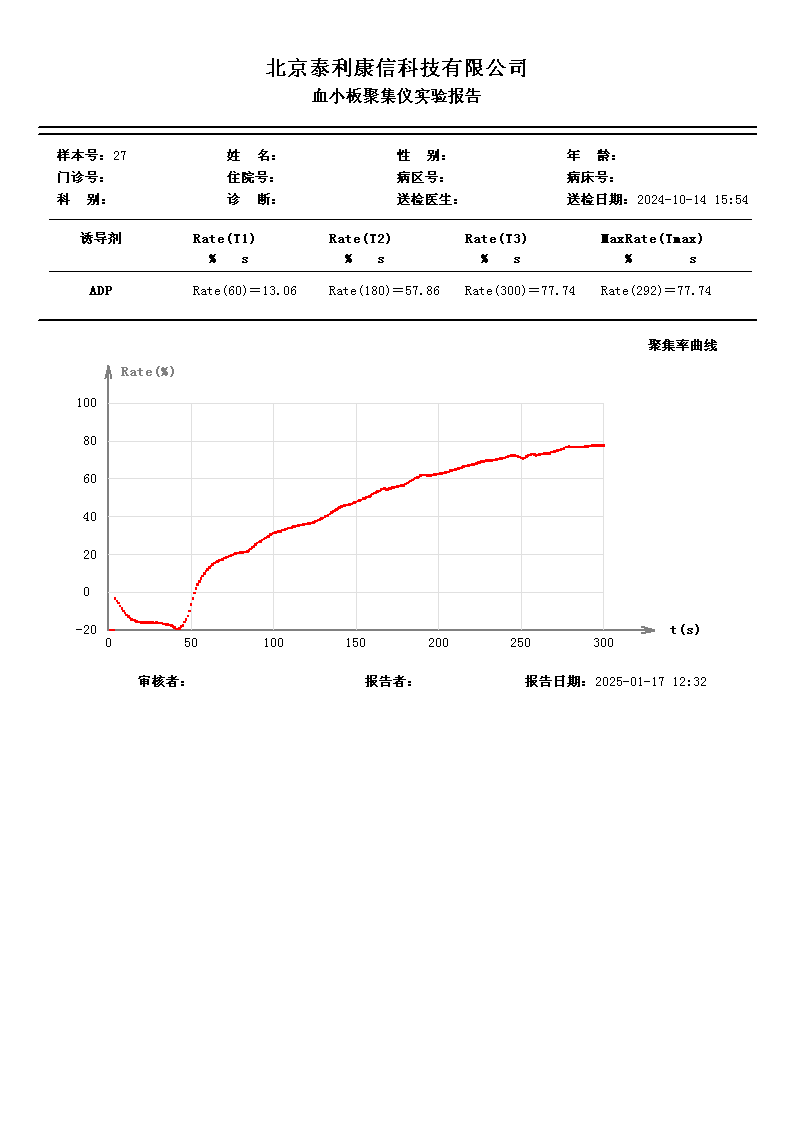
**

**The effective extract (EPI)**

**
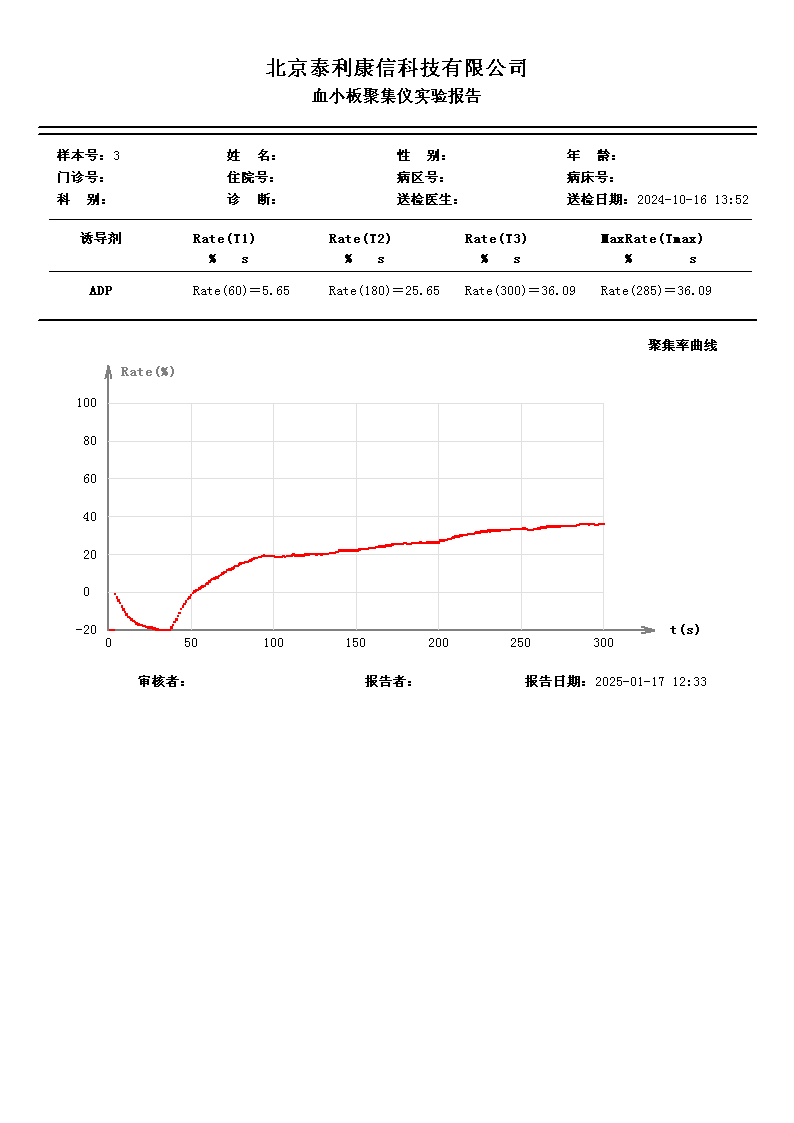
**

**Blank (PAF)**

**
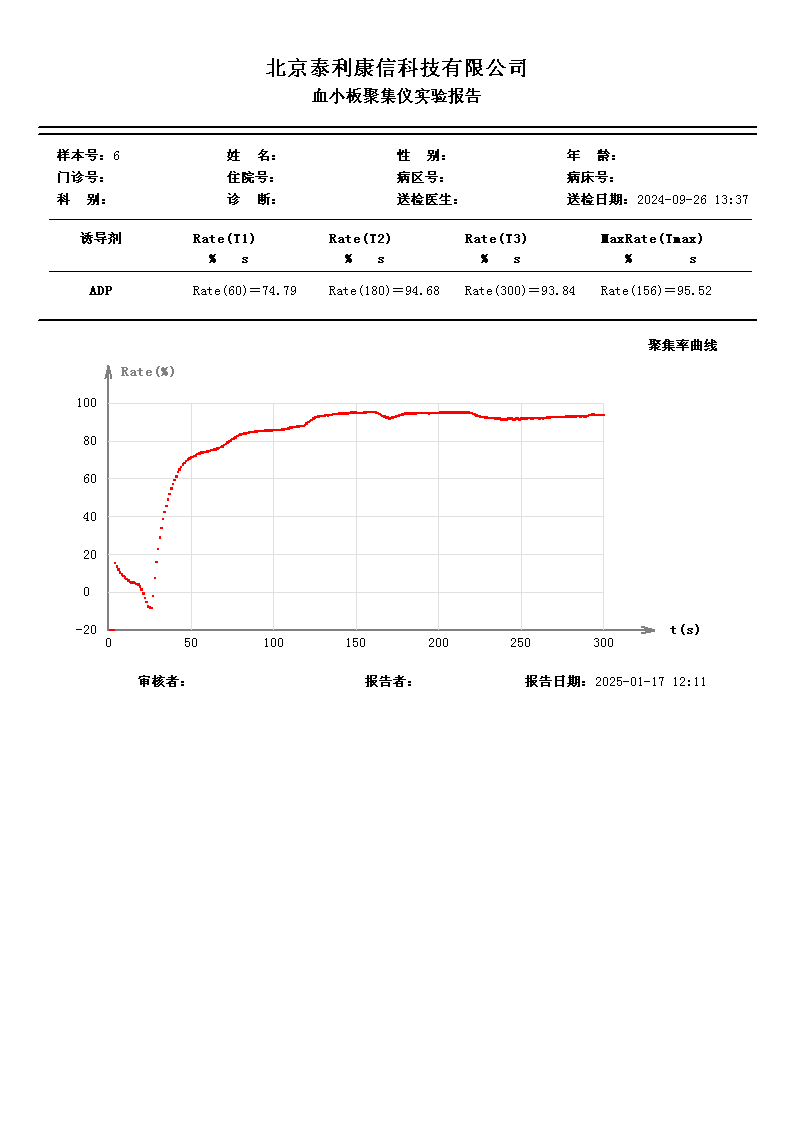
**

**The effective extract (PAF)**

**
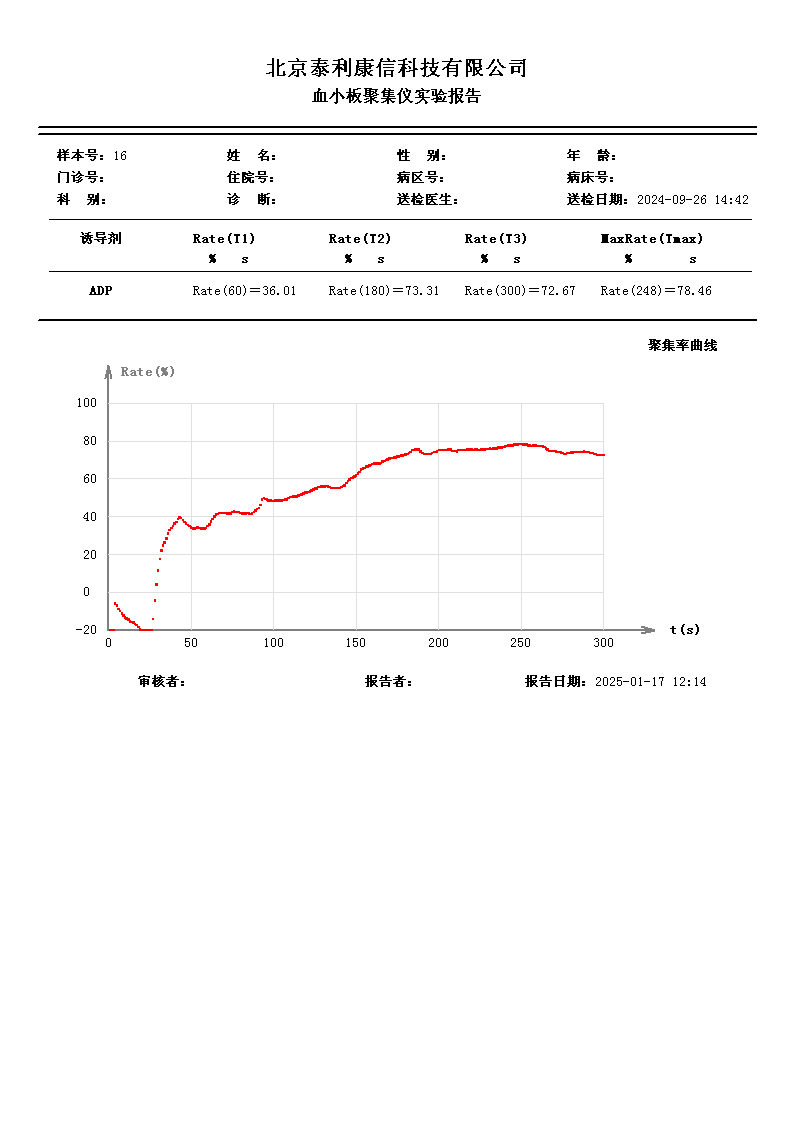
**

**CME (Thrombin)**

**
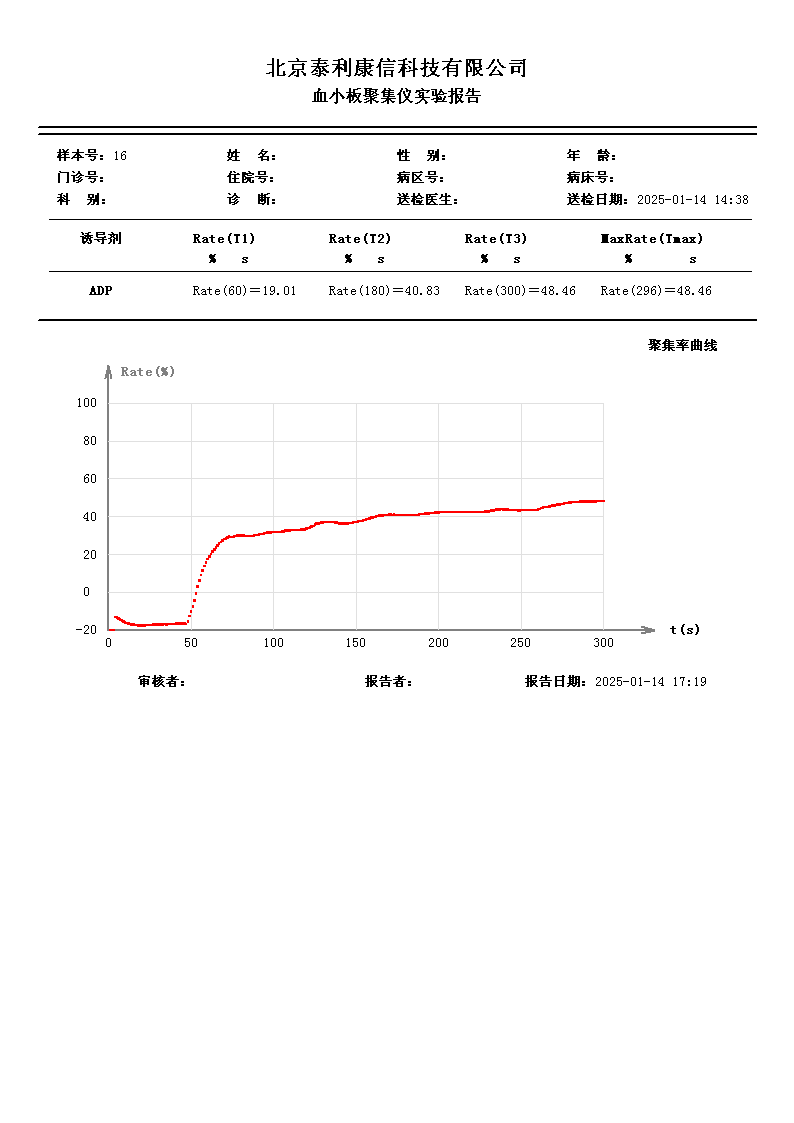
**

**JB (Thrombin)**

**
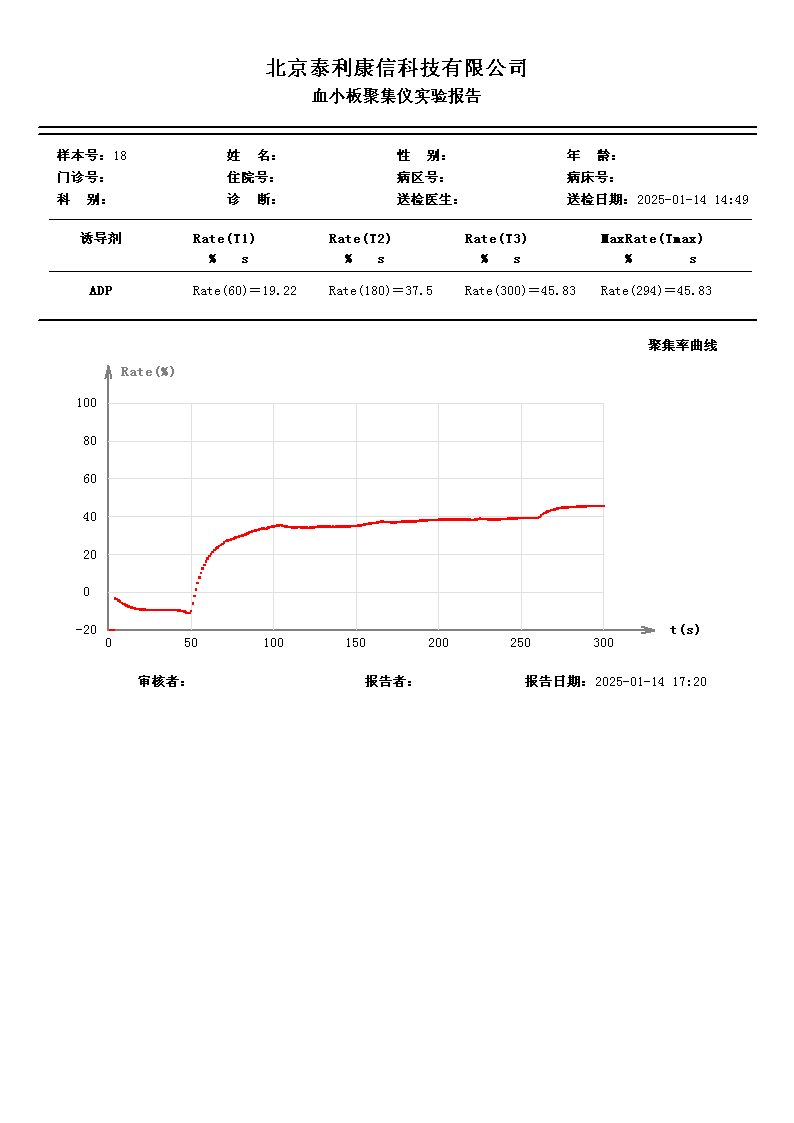
**

**Aspirin (Thrombin)**

**
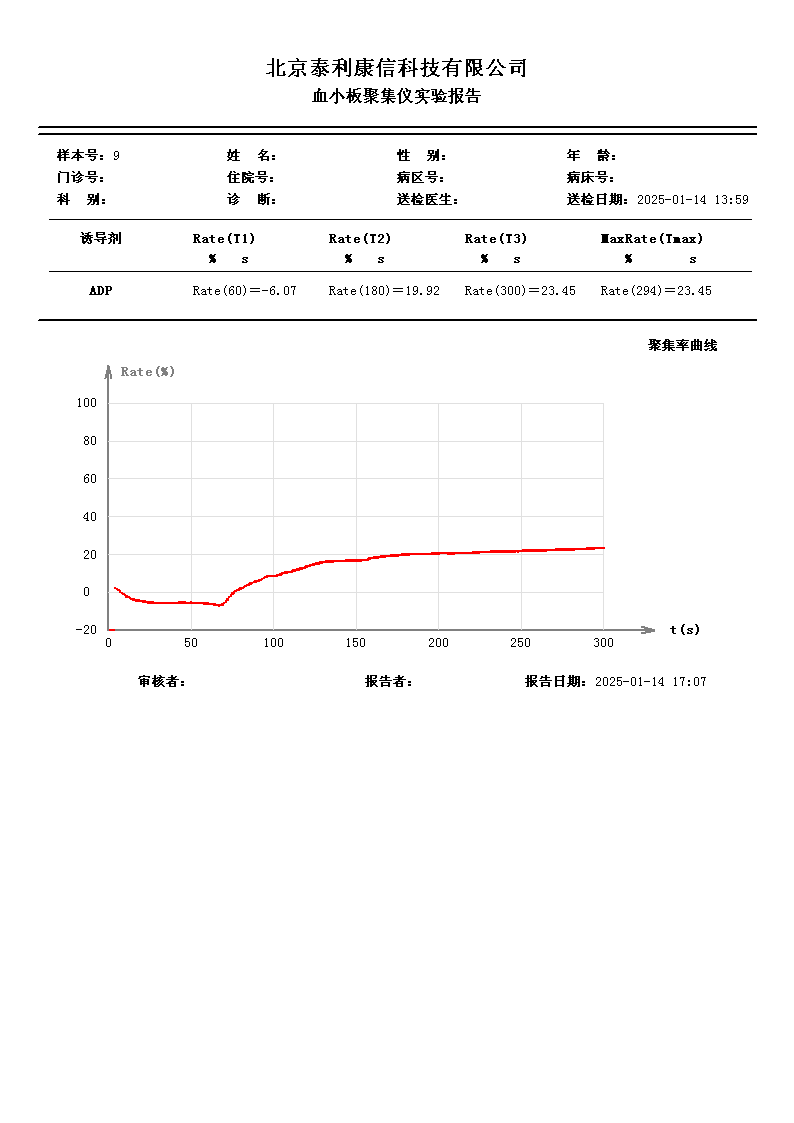
**

**Blank (Thrombin)**

**
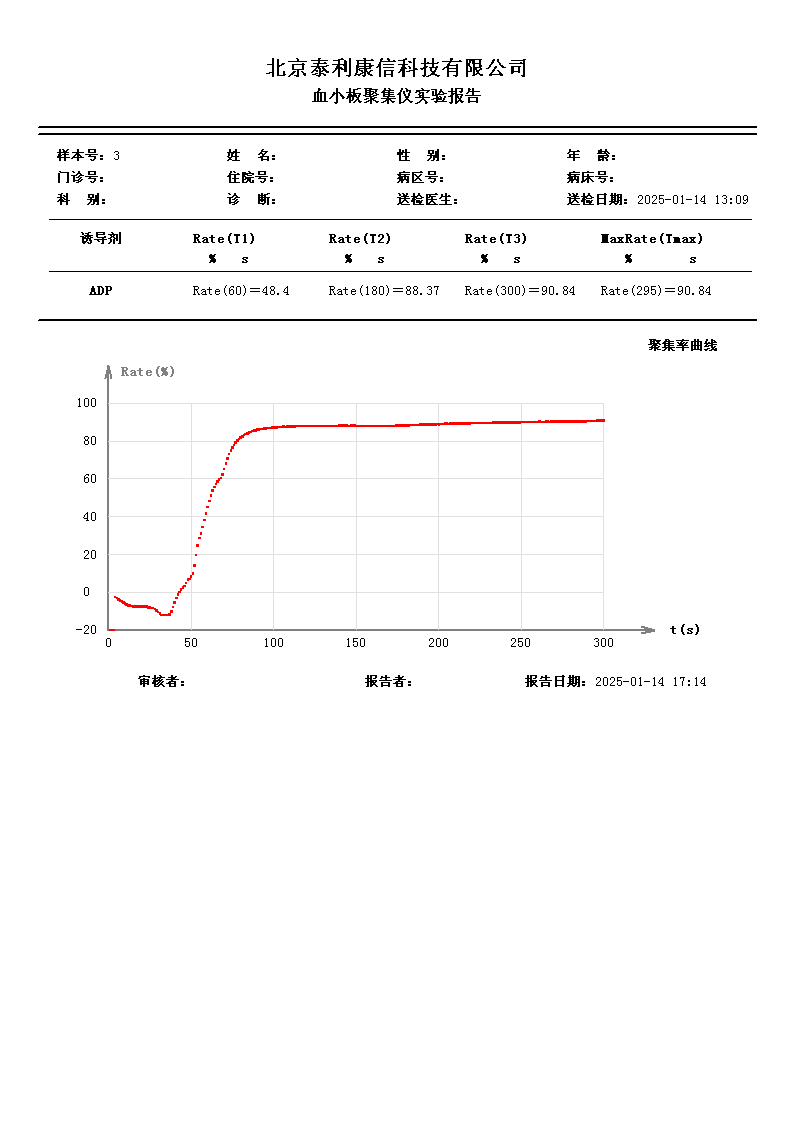
**

**The ethyl acetate extract (Thrombin)**

**
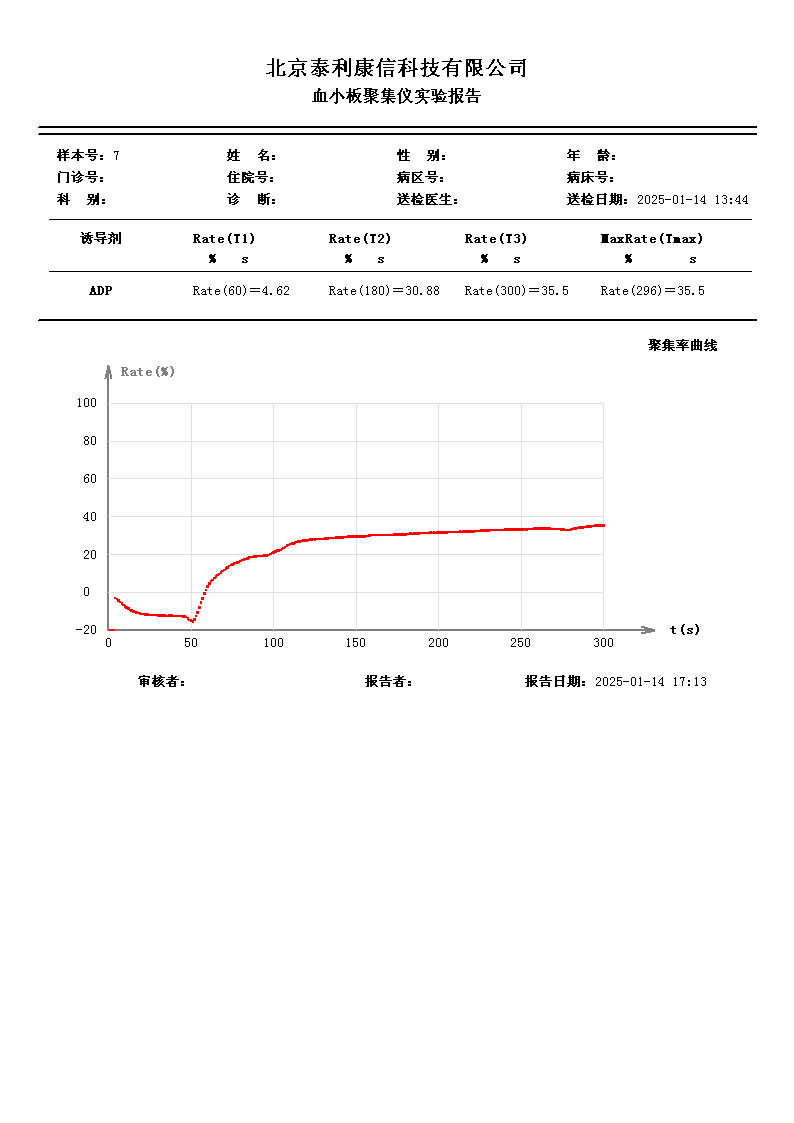
**

**The effective extract (Thrombin)**

**
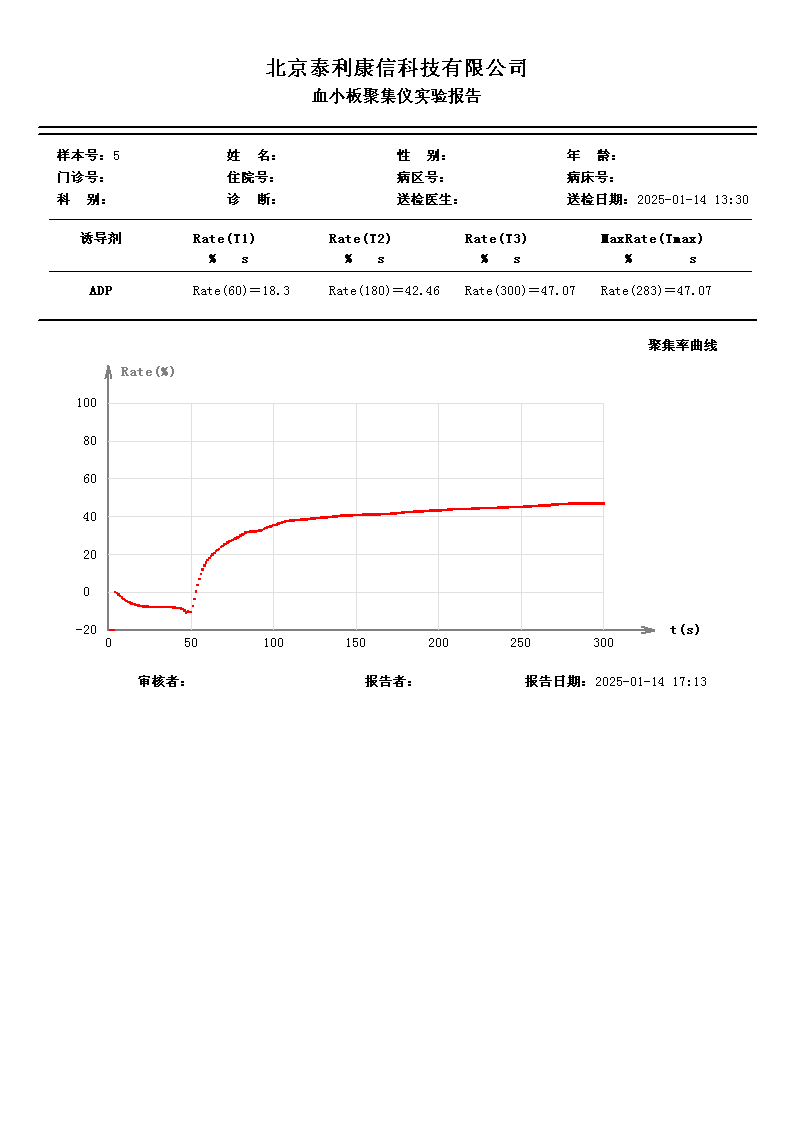
**
