# Supplementary figures and images for: Antithrombotic effect and plasma pharmacochemistry of Justicia Procumbens L
Source: PLoS One. 2025 Apr 30;20(4):e0321023. doi: 10.1371/journal.pone.0321023 (PMC12083875; doi:10.1371/journal.pone.0321023)

**S2 Fig. Platelet Aggregation Curve**


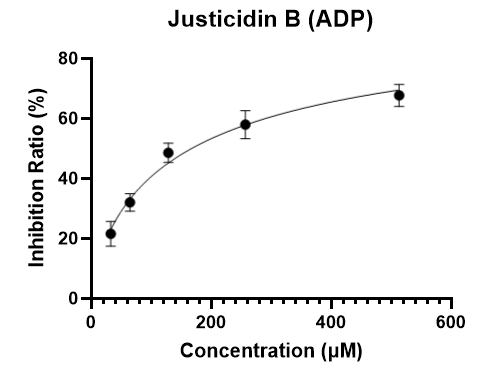


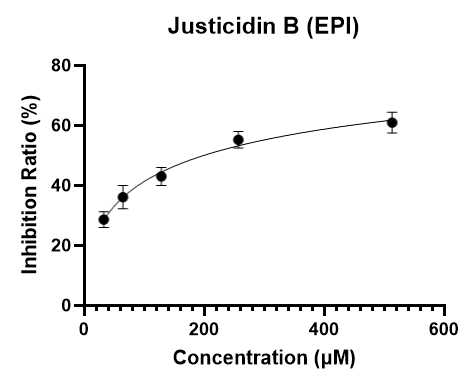

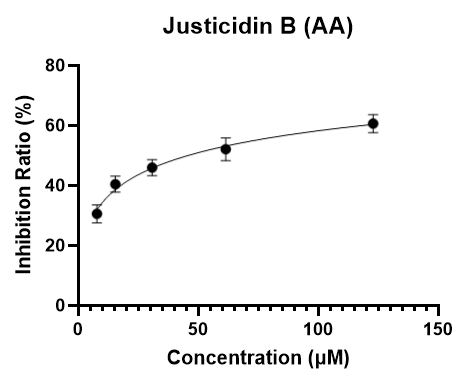

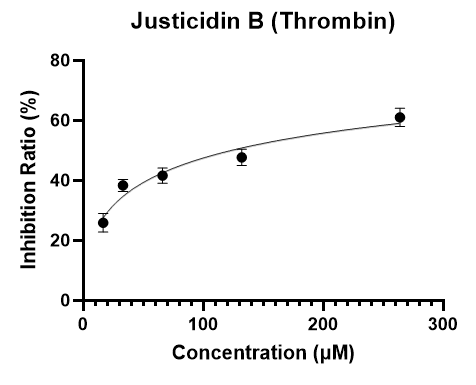


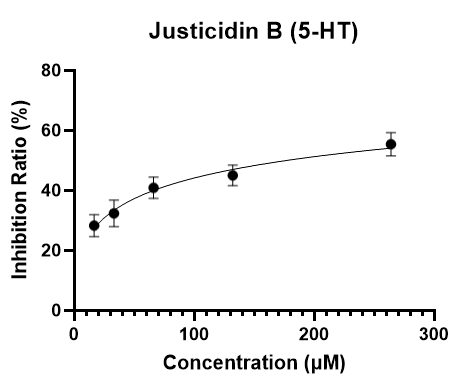

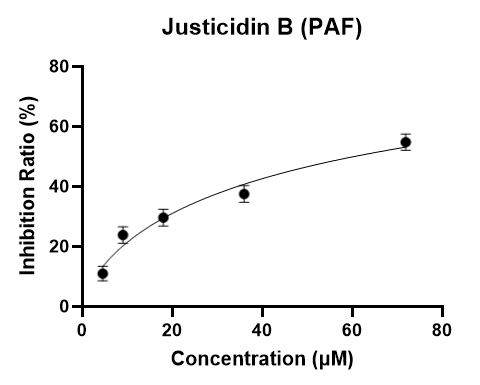


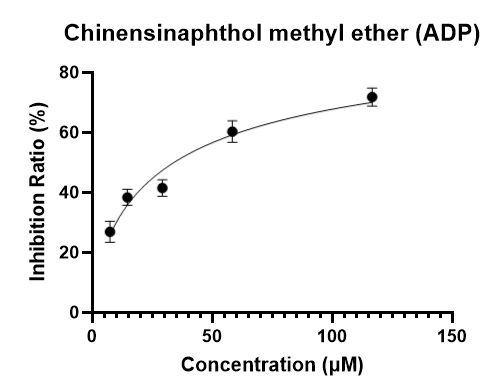


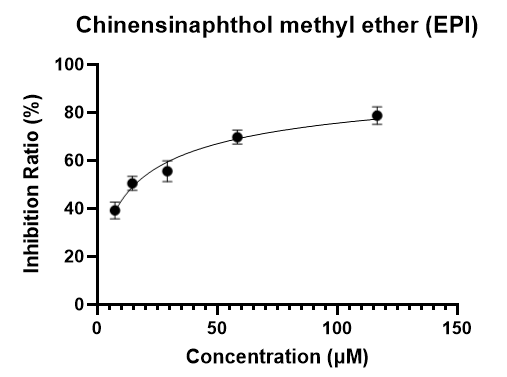


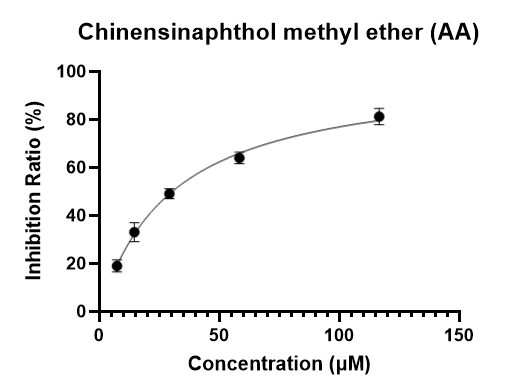


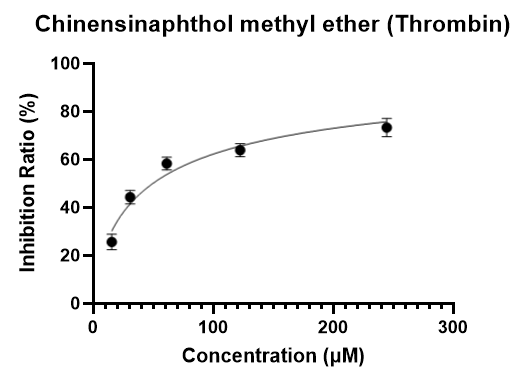


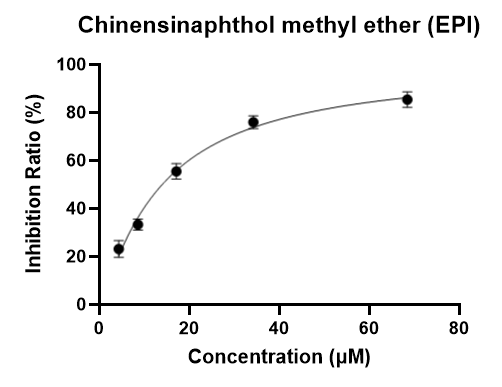


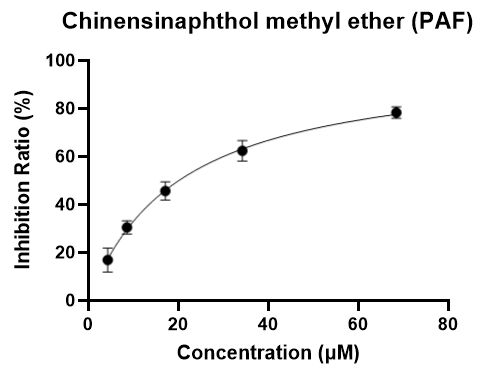


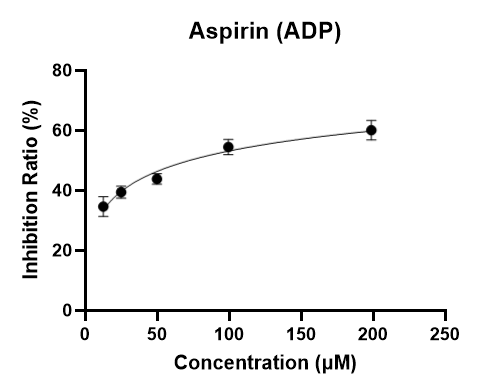


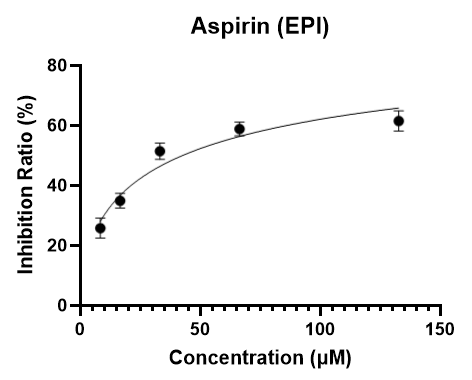


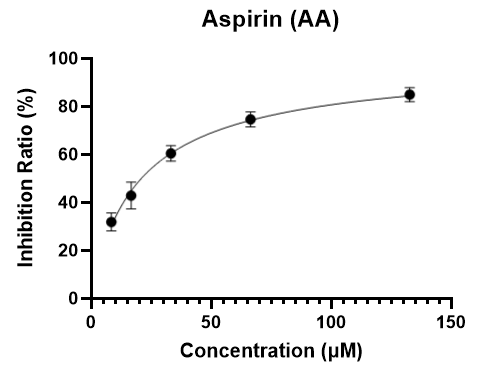


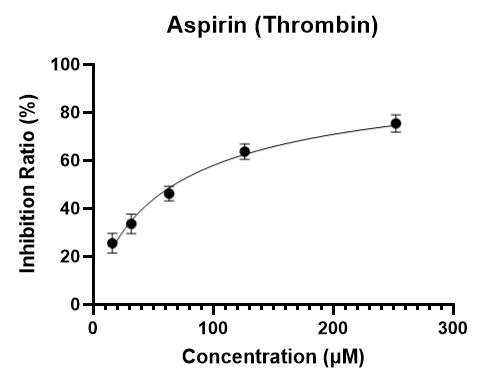


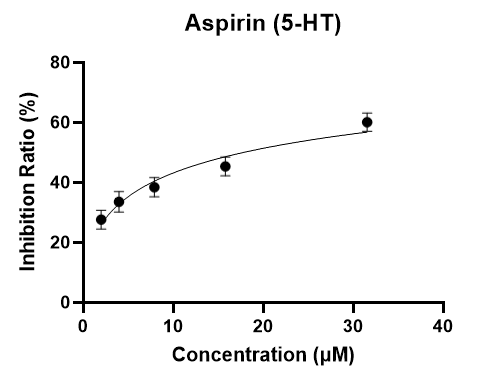


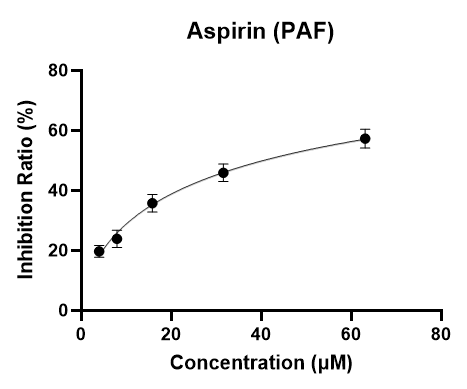


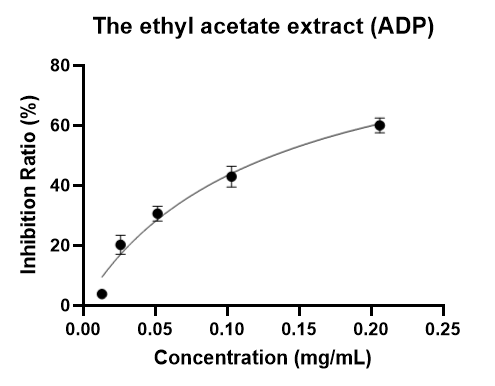


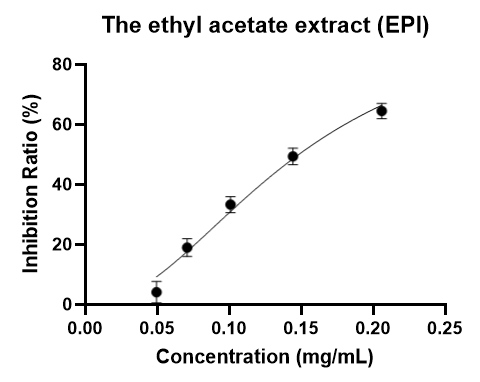


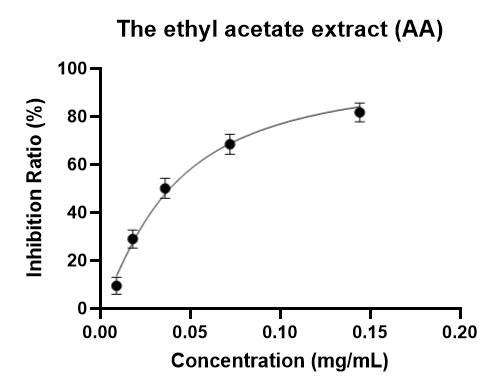


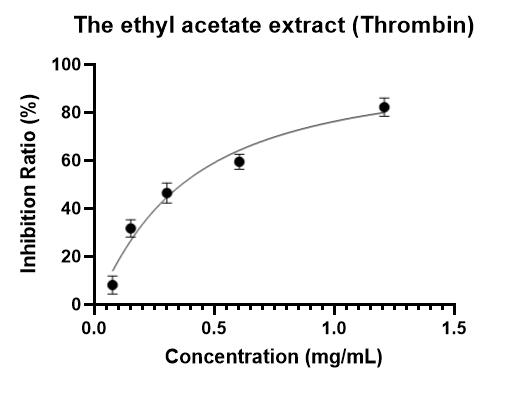


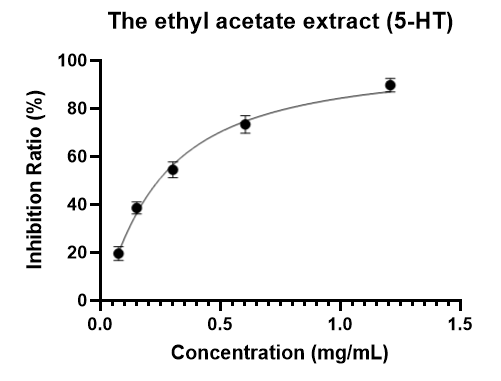


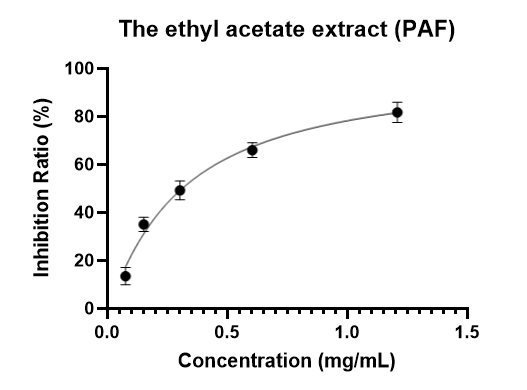


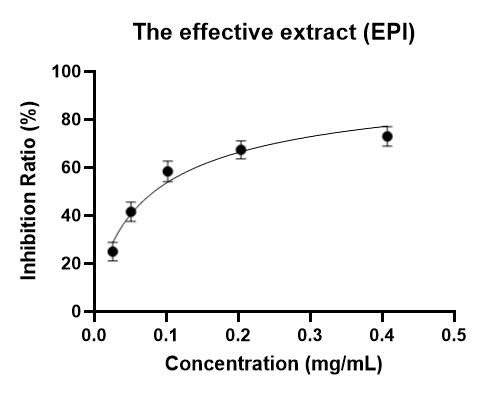


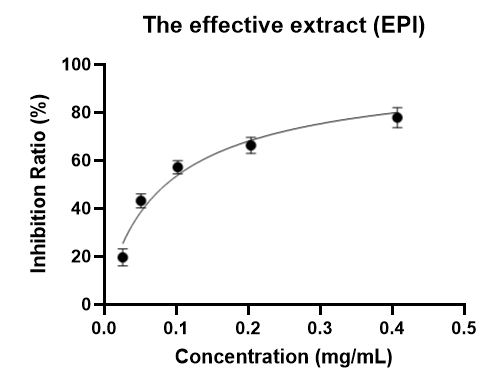


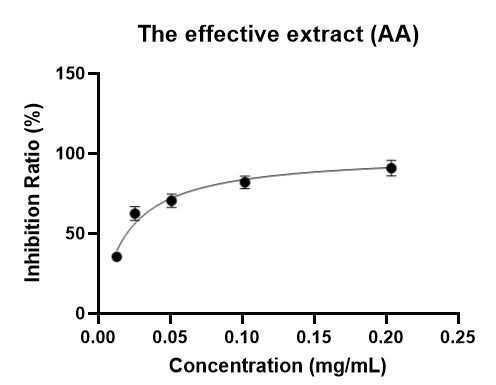


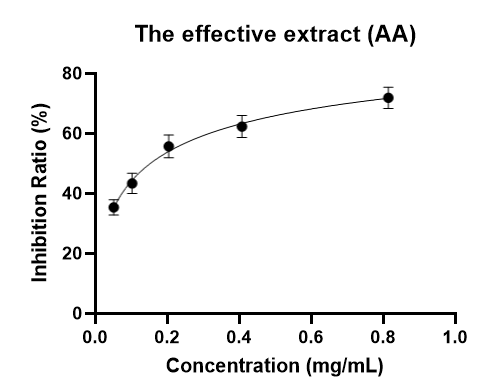


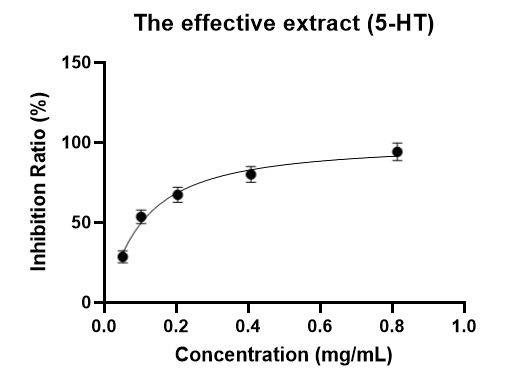


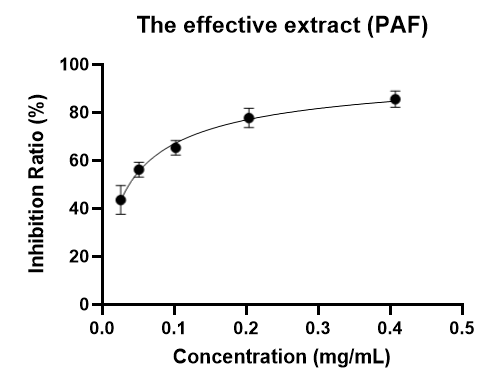

Supplement: S2 Fig — (DOC) [file pone.0321023.s006.doc]
